# Molecular Cytogenetic and Transcriptome Characterization of a Novel Wheat-*Thinopyrum intermedium* 2J(2D) Disomic Substitution Line with Resistance to Powdery Mildew

**DOI:** 10.3390/plants15193061

**Published:** 2026-10-07

**Authors:** Chen Wang, Chengzhi Jiang, Bingke Liu, Xiangyu Niu, Qingsong Qi, Yichao Sun, Jintao Yu, Zujun Yang, Deshun Feng

**Affiliations:** 1 State Key Laboratory of Wheat Improvement, College of Agronomy, Shandong Agricultural University, Tai’an 271018, China; 2 School of Life Science and Technology, University of Electronic Science and Technology of China, Chengdu 610054, China

**Keywords:** wheat (*Triticum aestivum* L.), *Thinopyrum intermedium*, disomic substitution line, powdery mildew, RNA-seq

## Abstract

Wheat powdery mildew, caused by the biotrophic pathogen *Blumeria graminis* f. sp. *tritici* (*Bgt*), is a destructive disease that causes severe yield losses in susceptible varieties, threatening global food security. Breeding resistant cultivars carrying novel resistance genes through wide hybridization represents a sustainable strategy for powdery mildew management. As a wild relative in the wheat tertiary gene pool enriched in diverse resistance genes, *Thinopyrum intermedium* serves as a valuable genetic resource for wheat improvement. A wheat–*Th. intermedium*-derived line SN6307 with powdery mildew resistance and multiple desirable agronomic traits was developed using wheat cultivar YN15 as the recipient crossing to *Th. intermedium*, and karyotypically characterized as a 2J(2D) disomic substitution line via non-denaturing fluorescence in situ hybridization (ND-FISH), Oligo-FISH painting, genomic in situ hybridization (GISH) and single-nucleotide polymorphism (SNP) analyses. To explore resistance genes in SN6307, we established a novel staining protocol for observing *Bgt* epiphytic structures and performed integrated second- and third-generation transcriptome sequencing on leaf epidermal tissues of SN6307 and YN15 after *Bgt* inoculation. Resistance reaction assays revealed that SN6307 exhibits high resistance and near immunity to *Bgt isolate E09* at the seedling stage. Significant differences in *Bgt*-responsive gene expression profiles and defense strategies were observed between SN6307 and YN15. Two *Th. intermedium*-derived leucine-rich repeat receptor-like kinase (LRR-RLK) family genes and several genes associated with plant–pathogen interactions were comprehensively analyzed and revealed that they coordinately modulate the multi-layered immune interaction network in SN6307. Furthermore, 24 novel *Th. intermedium* chromosome 2J-specific molecular markers were successfully developed, enabling efficient tracking of 2J chromatin in wheat populations derived from radiation mutagenesis. In conclusion, SN6307 represents novel germplasm for wheat disease-resistance breeding and further characterization of *Th. intermedium* functional genes.

## 1. Introduction

Wheat (*Triticum aestivum* L., 2n = 6x = 42, AABBDD) is the cereal crop with the largest cultivated area worldwide, feeding 35–40% of the global population [1]. Wheat powdery mildew, caused by the biotrophic pathogen *Blumeria graminis* f. sp. *tritici* (*Bgt*), is a devastating worldwide disease that leads to severe grain yield loss and threatens global food security [2]. Excessive reliance on chemical pesticides for disease control not only significantly increases production costs but also causes severe ecological damage. In contrast, breeding disease-resistant wheat varieties is the most economical and eco-friendly strategy to control powdery mildew epidemics.

Modern wheat has undergone long-term domestication and directional selection, resulting in an increasingly narrow genetic base with approximately 67% of the original genetic variation lost [3]. This severely limits the efficiency of wheat breeding for high yield and disease resistance. Fortunately, distant hybridization and chromosome engineering technologies provide a feasible solution to introduce beneficial genes from wild relatives into wheat, which have effectively enhanced genetic diversity and achieved remarkable results in developing disease-resistant varieties and improving wheat quality [4]. However, *Bgt* exhibits strong population genetic adaptability, a large effective population size, and a high mutation rate. As a result, single resistance genes widely used in production often lose effectiveness within a few years [5]. Therefore, current wheat breeding urgently requires the continuous development of widely applicable germplasm resources and the exploration of broad-spectrum, durable resistance genes from wild wheat relatives.

Wild relatives of wheat form a valuable germplasm resource pool with abundant disease resistance genes. *Thinopyrum intermedium* (2n = 6x = 42) is an important wild relative with multiple desirable traits: it has a deep root system, strong stress tolerance (including salt, drought, and cold resistance) [6], and resistance to powdery mildew and rust [7]. Additionally, it possesses superior nutritional quality, with higher dietary fiber, protein, and phenolic contents [8]. Notably, *Th. intermedium* possesses a complex polyploid genome that exhibits partial homology to wheat, facilitating successful hybridization.

As a core species for wheat improvement, the genome composition of *Th. intermedium* has long been a focus of cytogenetic research in the tribe *Triticeae*, but remains controversial due to highly repetitive sequences, historical interspecific hybridization events, and complex allopolyploid evolution [9]. Early genomic in situ hybridization (GISH) analysis proposed a genome formula of EeEeEbEbStSt, including two genomes similar to those of *Thinopyrum elongatum* (Ee or Je) and *Thinopyrum bessarabicum* (Eb or Jb), plus one genome distantly related to those of *Pseudoroegneria strigosa* (St) [10]. Subsequent phylogenetic analysis using expressed sequence tag-simple sequence repeat (EST-SSR) markers revised this to JvsJvsJrJrStSt, where Jvs and Jr are ancestral to modern Jb and Je, and the St genome is highly similar to that of Eurasian native diploid *Pseudoroegneria* species [11]. Recent advances in oligonucleotide fluorescence in situ hybridization (Oligo-FISH) suggested the Jvs genome is mainly derived from *Dasypyrum breviaristatum*, while J^r^ is closely related to *Thinopyrum* species [12]. Other studies based on ribosomal DNA (rDNA) evolution proposed two subgenomes from *Pseudoroegneria* and *Dasypyrum*, with the third from an ancient hybridization between *Thinopyrum* and *Aegilops* [13]. The latest genomic and cytological research updated the genome formula to JJVVStSt, where St is from *Pseudoroegneria*, J is closely related to *Aegilops*, and the unique V subgenome is contributed by *Dasypyrum* [9]. Meanwhile, a standard karyotype map has been constructed via non-denaturing fluorescence in situ hybridization (ND-FISH) and barley collinear region-based Oligo-FISH painting, providing a key tool for accurate identification of alien chromosomes in wheat backgrounds [14].

*Th. intermedium* serves as an important “tertiary gene pool” for improving wheat’s disease resistance, stress tolerance, and nutritional quality. For biotic stress, multiple broad-spectrum resistance genes have been identified: the aspartic protease gene *TiAP1* confers immunity in wheat by inactivating *Bgt* deacetylase [15]; the stripe rust resistance gene *YrT14* is finely mapped to chromosome 19 (homoeologous to 7J or 7Js) [16]; translocation lines carrying chromosomes 1St and 7Js are valuable for stem rust control [17]; and diploid accessions such as HS2-5 exhibit low fusarium head blight incidence [18,19]. For abiotic stress, the 7XL/7DS translocation line contains drought-induced proteins that enhance drought resistance stability [20], and its efficient Na^+^ exclusion mechanism supports salt-tolerant wheat breeding [21]. For quality improvement, its grain protein content is ~50% higher than that of wheat, with abundant dietary fiber, folic acid, and selenium [8,22].

However, the genetic compensation mechanisms of specific elite traits during distant hybridization, as well as the underutilization of disease resistance and stress tolerance genes from *Th. intermedium* alien chromatin, remain to be investigated. This study aims to create and precisely identify a novel wheat-*Th. intermedium*-derived line SN6307 with high powdery mildew resistance, superior quality, and salt tolerance; reveal transcriptional differences in response to *Bgt* infection; and exploit the genetic potential of *Th. intermedium*. These findings provide valuable germplasm resources and a scientific basis for breeding wheat varieties with improved powdery mildew resistance, with potential ecological and economic benefits.

## 2. Results

### 2.1. Genetic Composition of SN6307 and YN15 Revealed by GISH and Oligo-FISH

**Figure 1:**
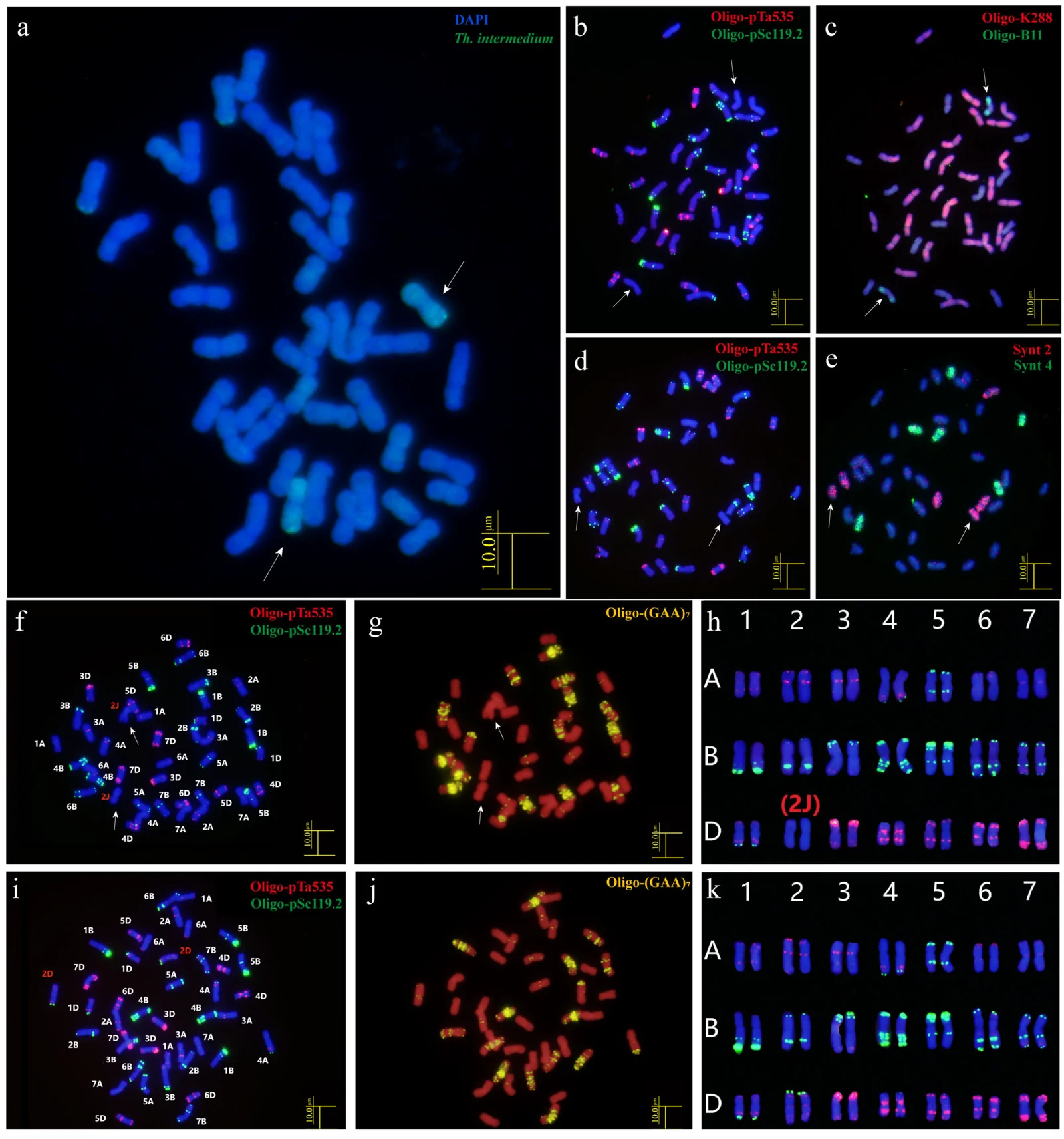
Karyotype analysis of SN6307 and YN15 using GISH, Oligo-FISH and Oligo-FISH painting: (**a**) Genomic in situ hybridization (GISH) of SN6307 using *Th. intermedium* genomic DNA as the probe (green) and YN15 genomic DNA as the blocker. (**b–g,i,j**) Sequential oligonucleotide fluorescence in situ hybridization (Oligo-FISH) and chromosome painting using Oligo-pTa535 (red) and Oligo-pSc119.2 (green) (**b,d,f,i**), Oligo-K288 (red) and Oligo-B11 (green) (**c**), Synt2 (red) and Synt4 (green) (**e**), and Oligo-(GAA_7_ (yellow) (**g,j**). (**h,k**) Karyotypes of SN6307 (**h**) and YN15 (**k**); numbers 1–7 indicate chromosome homoeologous groups, and A, B and D indicate wheat subgenomes. The “2J” label in h identifies the *Th. intermedium* chromosome pair replacing wheat chromosome 2D. Blue fluorescence indicates 4′,6-diamidino-2-phenylindole (DAPI) counterstaining; red probe signals may appear pink/purple upon overlap with blue fluorescence in the composite images but are designated as red throughout. White arrows indicate alien chromosomes. Scale bars = 10 μm.

Previously, we developed SN6306 via hybridization between common wheat cultivar YN15 and *Th. intermedium*—a wheat-*Th. intermedium* disomic alien addition line carrying a pair of alien chromosomes [15]. Subsequently, in advanced-generation populations derived from successive selfing, we identified SN6307, a genetically stable wheat-*Th. intermedium* disomic alien substitution line with a chromosome number of 2n = 42. To confirm the presence of alien chromosomes in SN6307, we combined genomic in situ hybridization (GISH), non-denaturing FISH (ND-FISH), and a *Thinopyrum*-specific Oligo probe (Oligo-B11) [23]. For GISH analysis, *Th. intermedium* genomic DNA served as the probe, and YN15 genomic DNA was used as the blocker. Results showed that SN6307 contained a pair of *Th. intermedium*-derived chromosomes (Figure 1a), with bright Oligo-B11 hybridization signals detected at both ends of these alien chromosomes (Figure 1c). Based on the hybridization signal profile of Oligo-B11, this pair of alien chromosomes was preliminarily identified as J-group chromosomes [24]. The remaining 40 wheat chromosomes consisting of 14 A-genome, 14 B-genome, and 12 D-genome chromosomes, with the absence of one pair of 2D chromosomes, were revealed by ND-FISH using probes Oligo-pSc119.2 and Oligo-pTa535 (Figure 1b,f) [25].

To further determine the homoeologous group and subgenome affiliation of the *Th. intermedium* chromosomes, Oligo-FISH painting was performed using two linkage group-specific probes [26]. These probes produced specific hybridization signals along the entire length of target chromosome arms: Synt2 generated strong red fluorescent signals on wheat chromosomes 2A, 2B, and the pair of alien chromosomes, while Synt4 produced strong green fluorescent signals on wheat chromosomes 4A, 4B, and 4D (Figure 1d,e). All chromosomes were further verified via ND-FISH with Oligo-pTa535, Oligo-pSc119.2, and Oligo-(GAA)_7_ probes [27] (SN6307: Figure 1f,g; YN15: Figure 1i,j).

Based on the combined results of sequential ND-FISH, Oligo-FISH Painting and GISH, the standard karyotype maps of SN6307 (Figure 1h) and YN15 (Figure 1k) were constructed. Collectively, these findings indicated that during successive selfing after hybridization, a pair of *Th. intermedium* 2J chromosomes replaced the pair of 2D chromosomes in YN15, leading to the formation of SN6307 as a wheat-*Th. intermedium* 2J(2D) disomic alien substitution line.

### 2.2. SNP Locus Variations in SN6307 Revealed by 55K Chip Analysis

To verify the genetic homology difference between SN6307 and YN15 at the molecular level, we genotyped young embryos of YN15 and SN6307 using the 55K wheat SNP chip (Zhongyu Jinbiao, Beijing, China). A systematic analysis focused on the types of SNP locus variation and Na genotype characteristics across all 21 wheat chromosomes (1A–7D).

For the parental line YN15, the mean proportion of missing SNP calls across chromosomes was 0.245% (median = 0.220%), with slight fluctuations among chromosomes (standard deviation [SD] = 0.173%). This indicates a low overall deletion rate with an even distribution across the genome (Table S3). In contrast, the disomic alien substitution line SN6307 had a mean proportion of missing SNP calls across chromosomes of 1.748% (median = 0.203%, SD = 6.672%), which was significantly higher than that of YN15. Notably, this increase was mainly driven by extreme abnormal loci on chromosome 2D (Table S3).

**Figure 2:**
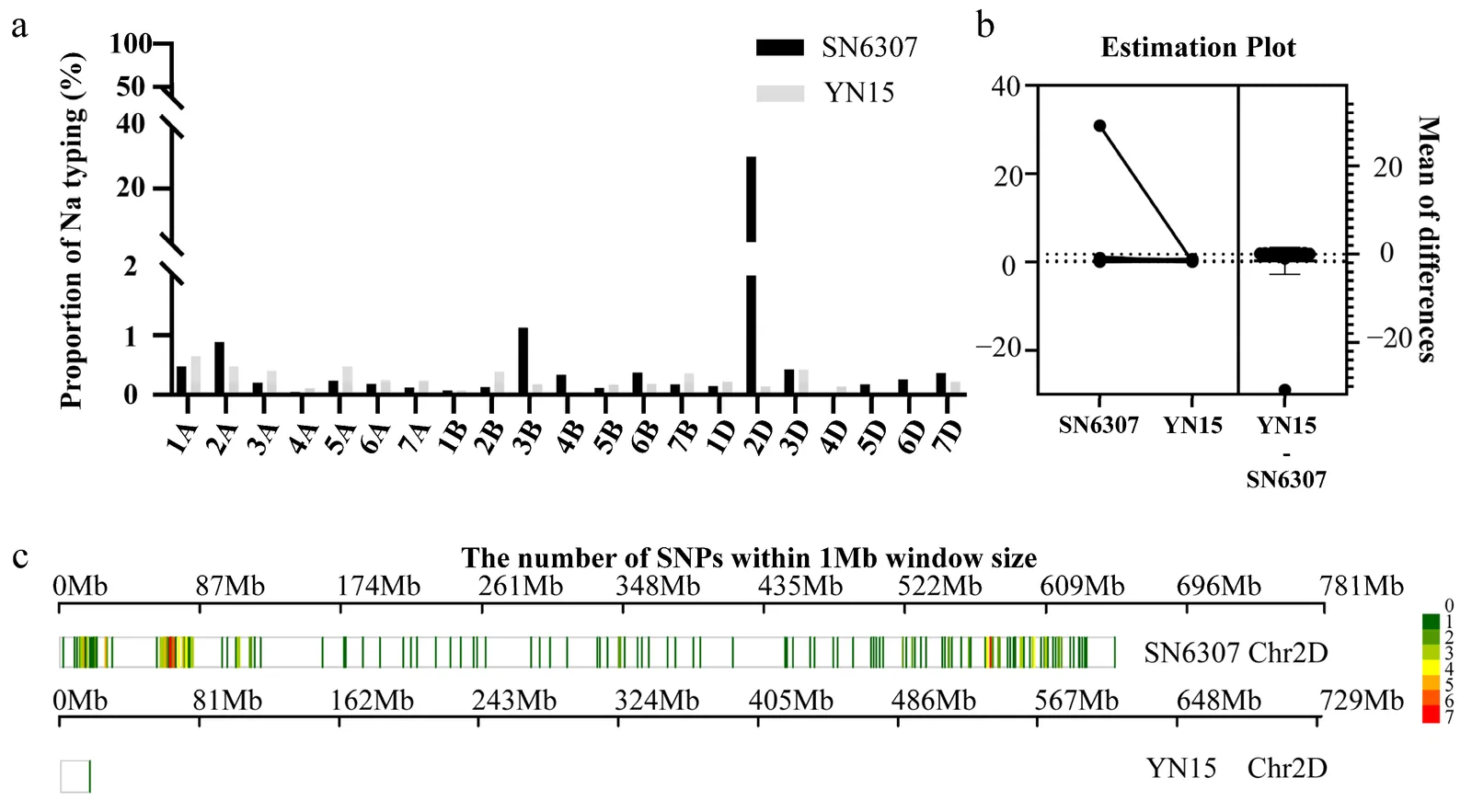
55K Wheat SNP Array Analysis: (**a**,**b**) SNP array analysis using the wheat 55K solid-phase chip was performed on the parental common wheat cultivar YN15 and the wheat–*Th. intermedium* 2J(2D) substitution line SN6307. Based on the standard marker information of the 55K solid-phase chip, the percentage of deleted loci on each chromosome relative to the total loci of that chromosome was calculated for both materials (**a**). The estimation plot (**b**) showed a significant difference in the proportion of Na genotype loci between SN6307 and YN15, which was attributed to chromosome 2D. The two dashed lines indicate zero on the original-data axis (left) and zero mean difference on the effect-size axis (right), respectively. The right-axis zero is aligned with the first-group mean, which is nonzero; therefore, the lines do not coincide. (**c**) The number and distribution of Na genotype loci across chromosome 2D were visualized using R software packages with a 1 Mb sliding window step. The scaled line segments represent chromosome lengths, and the rectangles below denote the chromosomal regions where Na genotype loci were detected.

When comparing SNP locus variation types and Na genotype ratios across the 21 chromosomes, the two materials showed highly significant differences specifically on chromosome 2D (Figure 2a,b). The Na genotype ratio on chromosome 2D in YN15 was only 0.1383%, a moderately low level across the genome with minimal deviation from other chip marker loci. In contrast, the Na genotype ratio of SN6307’s chromosome 2D reached 30.8437%—the only extreme outlier in the whole genome and 223-fold higher than that of YN15—with numerous locus deletion signals distributed across the entire chromosome (Figure 2c). To clarify the chromosomal distribution of this difference, we plotted the Na genotype distribution map with a 1 Mb window size (Figure S1). Results showed no obvious deletion enrichment in any interval of YN15’s chromosome 2D, while SN6307’s chromosome 2D exhibited strong deletion signals across all intervals, consistent with the extreme difference in Na genotype ratios.

Furthermore, genome-wide Na genotype analysis revealed conservative and specific characteristics between the two materials. Chromosomes 1B (both with a Na genotype ratio of 0.0694%) and 3D (both with a Na genotype ratio of 0.4264%) showed identical genotyping patterns, indicating high genetic homology and no significant genetic differentiation for these two chromosomes (Table S3). However, the distribution of D-genome chromosomes lacking Na genotype loci differed between materials: YN15 had no Na genotype loci on chromosomes 5D and 6D, while SN6307 lacked Na genotype loci only on chromosome 4D (Table S3). These results further confirm the genome-specific differences in SN6307 as an alien substitution line, providing direct molecular evidence for its identification and subsequent alien gene discovery.

### 2.3. Evaluation of Quality and Agronomic Traits

**Figure 3:**
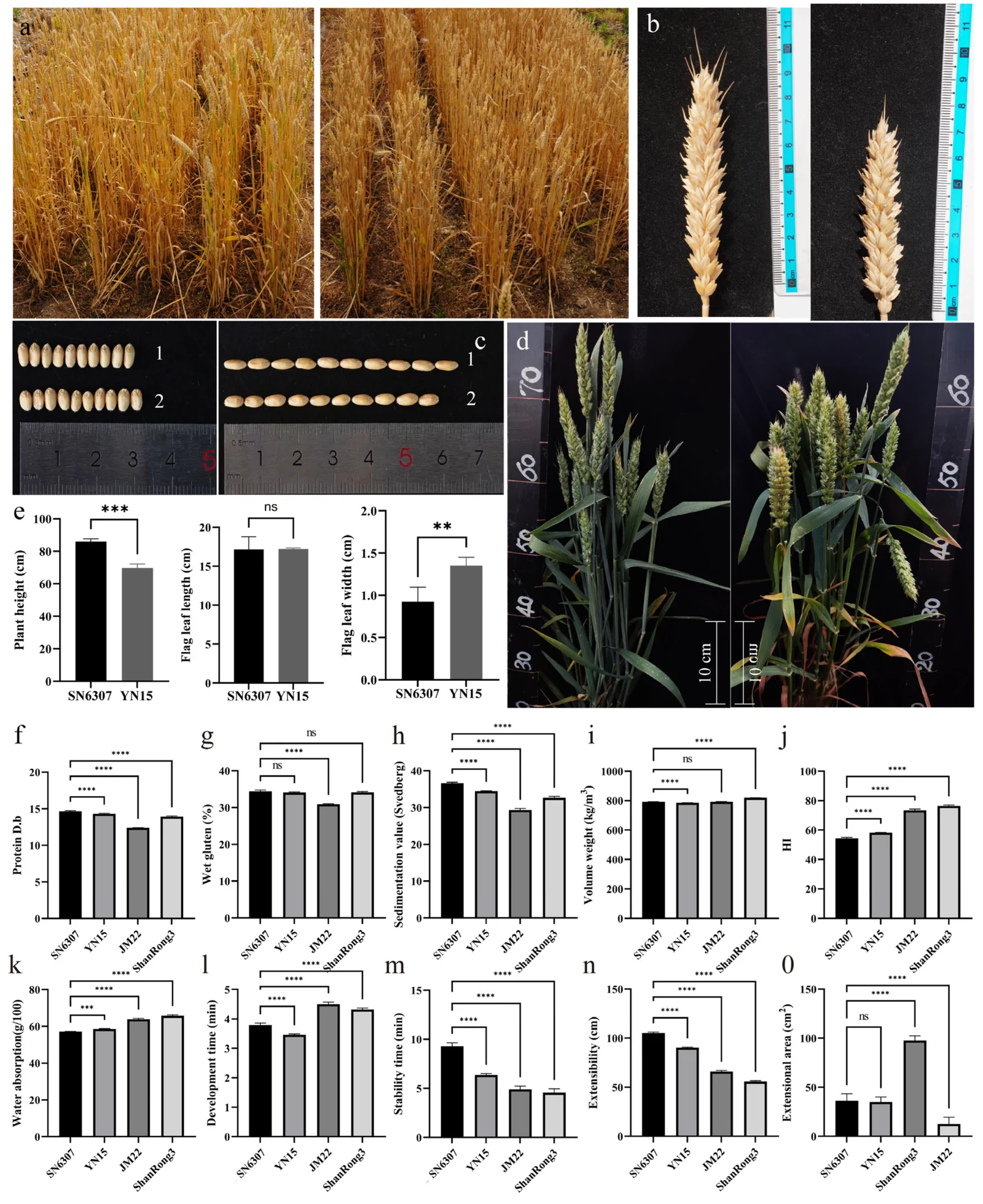
Analysis of agronomic traits and grain quality: (**a**) Population phenotypes of SN6307 (left) and YN15 (right) at the maturity stage. (**b**) Spike characteristics of SN6307 (left) and YN15 (right) at the maturity stage. (**c**) Grain morphology of SN6307 (1) and YN15 (2). (**d**) Individual plant phenotypes of SN6307 (left) and YN15 (right) at the grain-filling stage, scale bars in pannel d = 10 cm. (**e**) Plant height, flag leaf length and flag leaf width of SN6307 and YN15 at the grain-filling stage. (**f**–**o**) Analysis of grain quality traits including dry basis protein content (f) and other quality-related traits (**g**–**o**). Data are presented as mean ± SD (*n* ≥ 3). One-way ANOVA: ns, not significant; ** *p* < 0.01; *** *p* < 0.001; **** *p* < 0.0001.

Agronomic traits and grain quality of SN6307 and its parental line YN15 were systematically evaluated, showing that both materials exhibited a compact plant architecture and the same growth cycle (Figure 3a,e), and despite differing only in one pair of chromosomes (2D replaced by 2J), significant differences were observed in plant morphology and certain grain quality traits between the two lines (Figure 3d). Compared with YN15, SN6307 showed significantly longer spikes at maturity (*p* < 0.05); its grains were narrower and longer (Figure 3b,c), and it had a higher plant height, while flag leaf length showed no significant difference and flag leaf width was significantly reduced (*p* < 0.05) (Figure 3e), whereas hundred-kernel weight based on five random replicates showed no significant difference between the two lines (Table S4). In terms of grain quality, SN6307 either did not differ significantly from or outperformed the parental line YN15 and control cultivar JM22 in several key quality traits (Figure 3f–o), specifically exhibiting superior performance in nutritional quality-related traits (e.g., protein content) and processing quality-related traits (e.g., gluten index) while other traits (e.g., starch content) remained comparable to the controls.

Collectively, these results indicate that the 2J(2D) chromosome substitution had no significant adverse effect on the overall plant phenotype and grain quality of wheat; on the contrary, the introgression of the alien 2J chromosome improved certain quality traits of SN6307, providing valuable biological materials and experimental evidence for the subsequent isolation of key functional loci via radiation mutagenesis.

### 2.4. Investigation of Salt Tolerance and Bgt Resistance

**Figure 4:**
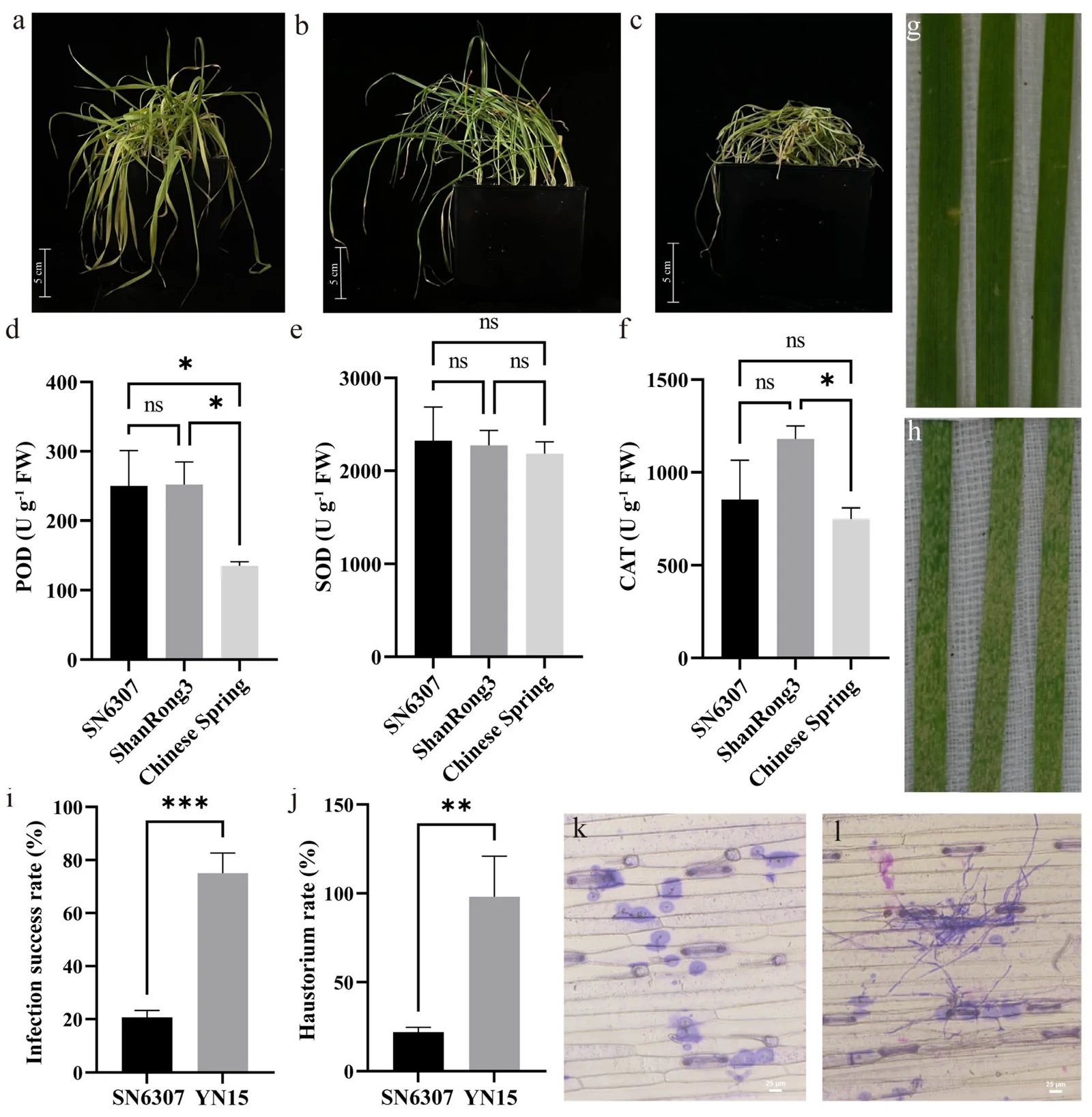
Analysis of salt tolerance and powdery mildew resistance in SN6307: (**a**–**c**) Phenotypes of SN6307 (**a**), salt-tolerance control lines ShanRong3 (**b**) and Chinese Spring (CS) (**c**) after 7 days of culture in Hoagland’s nutrient solution containing 250 mmol/L NaCl. Forty-eight plants per material were used. (**d**–**f**) Measurements of peroxidase (POD, **d**), superoxide dismutase (SOD, **e**), and catalase (CAT, **f**) activities in seedlings of the three wheat lines treated with 200 mmol/L NaCl in Hoagland’s nutrient solution for 7 days. Data are presented as mean ± SD (n ≥ 3). One-way ANOVA: ns, not significant; * *p* < 0.05; ** *p* < 0.01; *** *p* < 0.001. (**g**,**h**) Lesion phenotypes of detached leaves inoculated with *Bgt* conidia for 7 days: SN6307 (**g**) and YN15 (**h**). (**i**) Infection success rate in SN6307 and YN15. Total pixel count and lesion pixel count of each treated leaf were measured using ImageJ (v 1.54p) to calculate the proportion of the lesion area. (**j**) Counts of infected cells and haustoria were used to calculate the haustorium formation rate in infected cells. Data are presented as mean ± SD (n ≥ 3). Student’s t-test: ns, not significant; * *p* < 0.05; ** *p* < 0.01; *** *p* < 0.001. (**k**,**l**) Phenotypic differences in leaves from SN6307 (**k**) and YN15 (**l**) at 72 h post-inoculation with *Bgt* conidia, visualized by modified CBB-R250 staining. Scale bar = 25 μm.

*Th. intermedium* harbors excellent disease and stress resistance genes, so we evaluated the improved salt tolerance of SN6307 conferred by the 2J(2D) chromosome substitution, using the salt-tolerant cultivar ShanRong3 and the salt-sensitive cultivar Chinese Spring as controls. After 7 days of treatment in Hoagland nutrient solution supplemented with 250 mmol/L NaCl, salt tolerance was assessed by measuring biomass, phenotypic performance, and physiological indices. Results showed that SN6307 had significantly higher biomass than both ShanRong3 and CS (Figure 4a–c); although its leaves turned light green, the activities of salt stress-responsive enzymes ( peroxidase (POD), superoxide dismutase (SOD), and catalase (CAT)) were not significantly different from those of ShanRong3, and its POD activity was significantly higher than that of CS (Figure 4d–f), demonstrating that SN6307 exhibits good salt tolerance at the seedling stage.

In our previous study, YN15 was found to be susceptible to *Bgt* isolate *E09*, so we inoculated seedling leaves of SN6307 with *Bgt E09* conidia to explore powdery mildew resistance genes introduced by *Th. intermedium* chromosome 2J. Inoculation assays on YN15 and SN6307 showed that YN15 was susceptible (infection type (IT) = 3–4) with leaves fully covered by spores and necrotic spots, while SN6307 exhibited high resistance to powdery mildew (IT = 0–1) with no visible spore formation and healthy leaves (Figure 4g,h). Coomassie Brilliant Blue staining (Figure 4k,l) further confirmed that the spore infection success rate (Figure 4i) and haustorium formation rate (Figure 4j) of SN6307 were extremely significantly lower than those of YN15. Collectively, these results indicate that chromosome 2J may contain novel powdery mildew (*Pm*) resistance genes against the prevalent *Bgt* isolate *E09*, which points to a potential new *Pm* resistance locus on this chromosome.

### 2.5. Analysis of the Effects of Th. intermedium 2J Chromosome Substitution on Gene Expression in Wheat in Response to Bgt Infection

Transcriptome sequencing was performed to explore how chromosome 2J of *Thinopyrum intermedium* affects wheat gene expression upon *Bgt* infection. Third-generation full-length transcriptome sequencing of SN6307 generated 18,143 non-redundant full-length transcripts. These transcripts were used to systematically assess changes in genome expression in wheat caused by alien gene introgression and alien chromosome introduction. We further integrated these data with short-read RNA-seq (RNA sequencing) datasets from SN6307 and YN15 sampled at 12, 24 and 48 hpi with *Bgt*.

**Figure 5:**
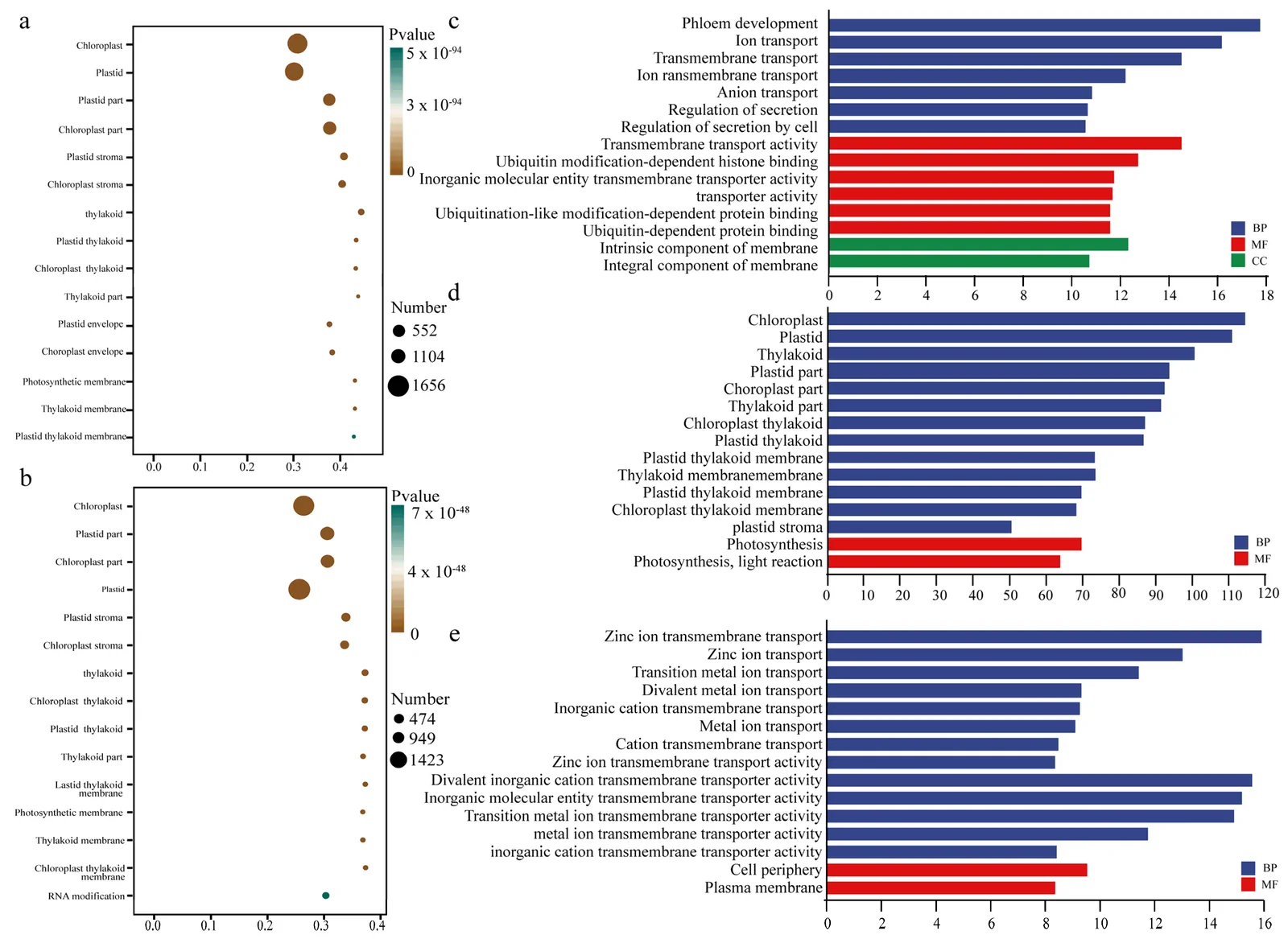
Transcriptome characteristics of SN6307 and YN15 in response to powdery mildew infection: (**a**,**b**) Gene Ontology (GO) enrichment analysis of up-regulated DEGs in SN6307 (**a**) and YN15 (**b**) at 12 hpi with *Bgt* conidia relative to the mock-inoculated control (top 15 enriched terms). In panels a and b, bubble size indicates the number of differentially expressed genes associated with each GO term, and bubble color indicates the enrichment adjusted p-value, with brown representing lower values and green representing higher values. (**c**–**e**) GO enrichment analysis of up-regulated DEGs in SN6307 compared with YN15 at 12 (**c**), 24 (**d**) and 48 hpi (**e**) with *Bgt* conidia (top 15 enriched terms). The ordinate represents the GO term, and the abscissa represents -log_10_ of the adjusted *P*-value for GO enrichment.

Pearson correlation analysis revealed clear time-dependent clustering across all samples (Figure S2). SN6307 and YN15 shared highly similar gene expression profiles before *Bgt* inoculation, whereas obvious transcriptional divergence emerged at 12 and 24 hpi (Figure S3). Gene Ontology (GO) enrichment on upregulated genes (relative to uninoculated controls) showed markedly stronger enrichment in SN6307 (Figure 5a) than in YN15 (Figure 5b) at the early infection stage (12 hpi). Genes from both genotypes were jointly enriched in functional terms linked to chloroplast, plastid and thylakoid components, demonstrating that photosynthetic organelles serve as core transcriptional response targets in wheat during early *Bgt* attack. Nonetheless, more SN6307 genes were associated with chloroplast- and plastid-related categories, with significantly higher enrichment scores for multiple thylakoid-associated GO terms compared with YN15.

Targeted pathway analysis further confirmed prominent plastid/chloroplast envelope enrichment exclusively in SN6307. These subcellular structures mediate transmembrane signal transduction and transport of defensive metabolites including salicylic acid and jasmonic acid precursors. By contrast, YN15 showed specific enrichment in RNA modification pathways, implying susceptible wheat lines may activate post-transcriptional regulation to compensate for impaired defense responses.

GO analysis of differentially expressed genes (DEGs) commonly upregulated in the two genotypes uncovered a stepwise defensive strategy uniquely adopted by SN6307 across all three tested time points. At 12 hpi (initial infection stage), defense activation relied mainly on phloem development, ion transmembrane transport and ubiquitin-dependent protein binding (Figure 5c). At 24 hpi (peak defense stage), profound transcriptome remodeling occurred, with upregulated genes strongly enriched in chloroplast components and photosynthesis pathways; this identifies chloroplasts as central hubs for metabolism and signal transduction (Figure 5d). At 48 hpi (late infection stage), functional priority shifted toward metal ion homeostasis and reinforcement of the plasma membrane and peripheral cell structures. This sequential regulatory shift outlines the layered resistance mechanism of SN6307: defense initiates via early signal perception and protein trafficking, progresses to large-scale metabolic reprogramming, and finally establishes resistance through homeostatic maintenance and structural fortification (Figure 5e).

### 2.6. Mining and Functional Characterization of Core Resistance Genes Based on Dynamic Transcriptional Clustering

K-means clustering was applied to classify dynamic gene expression patterns of SN6307 across sequential *Bgt* infection stages, partitioning all expressed genes into nine distinct clusters (Figure S4). Genes in Clusters 1, 3 and 6 were persistently upregulated as infection proceeded, while Clusters 2, 4 and 9 displayed significant downregulation or an early expression burst followed by a sharp decline. Conversely, Clusters 5 and 8 underwent transient upregulation between 12 and 24 hpi before gradual expression reduction, and Cluster 7 showed an initial expression suppression followed by expression recovery. These discrete expression clusters regulate diverse biological processes such as plant immune signaling and secondary metabolite biosynthesis, jointly constructing a multi-layered regulatory network against *Bgt* invasion in wheat.

**Figure 6:**
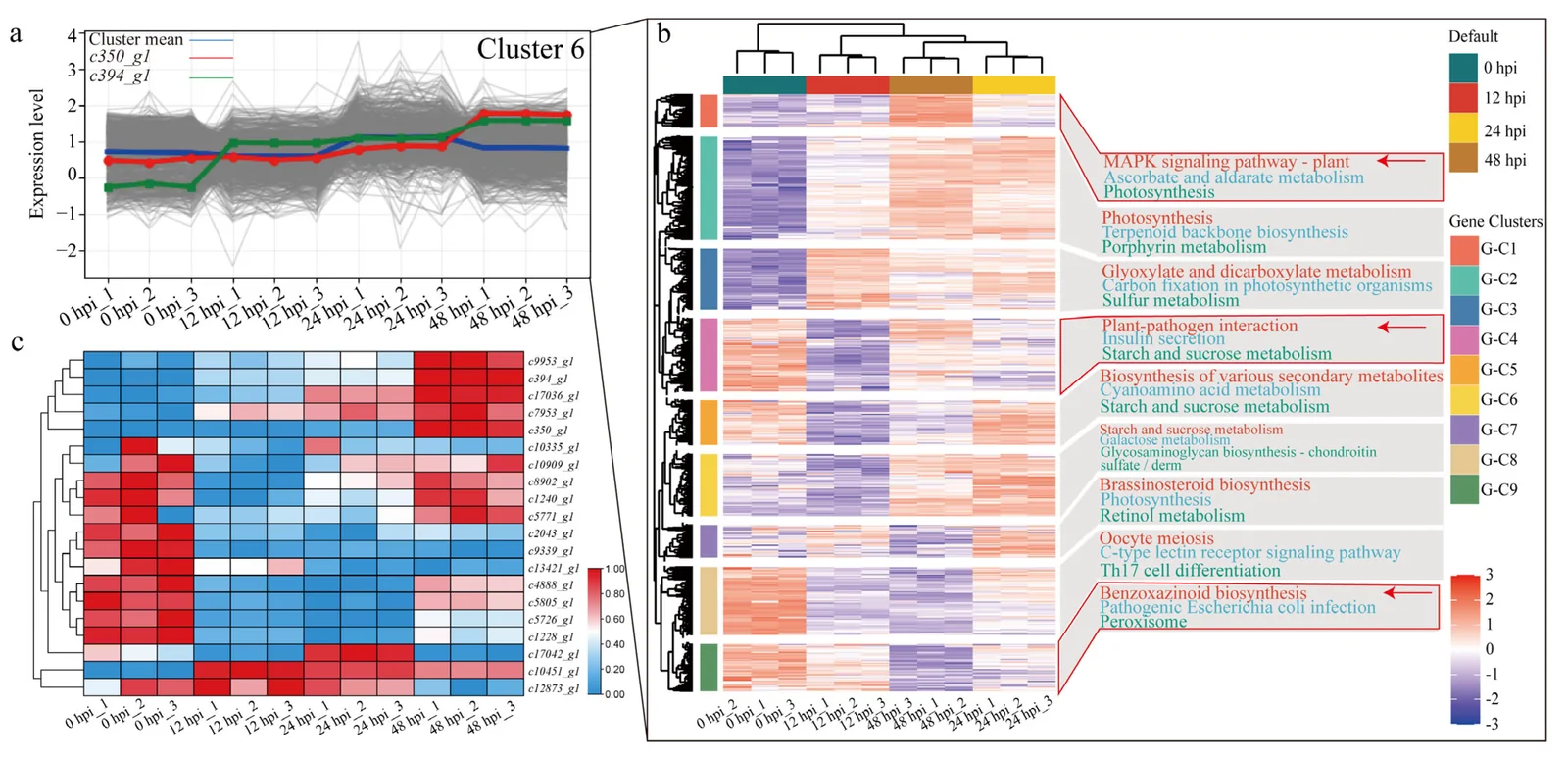
Expression profiling of candidate resistance genes derived from *Th. intermedium* chromosome 2J: (**a**) Temporal expression dynamics of all genes in Cluster 6 at four time points (0, 12, 24, 48 hpi) following *Bgt* inoculation. Colored lines represent the mean expression of target transcripts *c350_g1* and *c394_g1*. Thin gray lines represent the expression trajectory of individual transcripts within each cluster, while solid blue lines indicate the mean expression trend of all transcripts in the corresponding cluster. (**b**) K-means clustering heatmap of global differentially expressed genes (DEGs) and KEGG enrichment results for Cluster 6; red arrows label three core defense-related metabolic pathways significantly enriched in this gene cluster. (**c**) Expression abundance heatmap of all genes belonging to the three KEGG pathways highlighted by red arrows in panel b.

Notably, transcripts within Cluster 6 displayed steadily rising expression as the disease progressed (Figure 6a). This cluster contained 754 genes with prominent time-dependent transcriptional dynamics. Early-upregulated subclusters G-C4, G-C5, G-C6 and G-C8 were predominantly enriched in plant–pathogen interaction, secondary metabolite biosynthesis and carbohydrate metabolism pathways, functioning in pathogen recognition and early defensive responses. Mid-stage induced G-C3 contributed to carbon and sulfur metabolic reprogramming to supply substrates for subsequent defense execution. Late-activated subclusters G-C1, G-C2, G-C7 and G-C9 sustained long-term disease resistance and stress repair via mitogen-activated protein kinase (MAPK) signaling, photosynthesis modulation, brassinosteroid signaling and benzoxazinoid biosynthesis pathways (Figure 6b). Moreover, 20 core genes participating in pivotal plant–pathogen interaction pathways were screened from Cluster 6, among which 15 functional genes and 5 long non-coding RNAs (Table S5) were annotated as disease-resistance candidates (Figure 6c). Notably, among the 20 genes screened from this cluster, 14 were annotated as originating from *Th. intermedium*, and the remaining 6 are highly conserved between wheat and *Th. intermedium* (Table S5).

**Figure 7:**
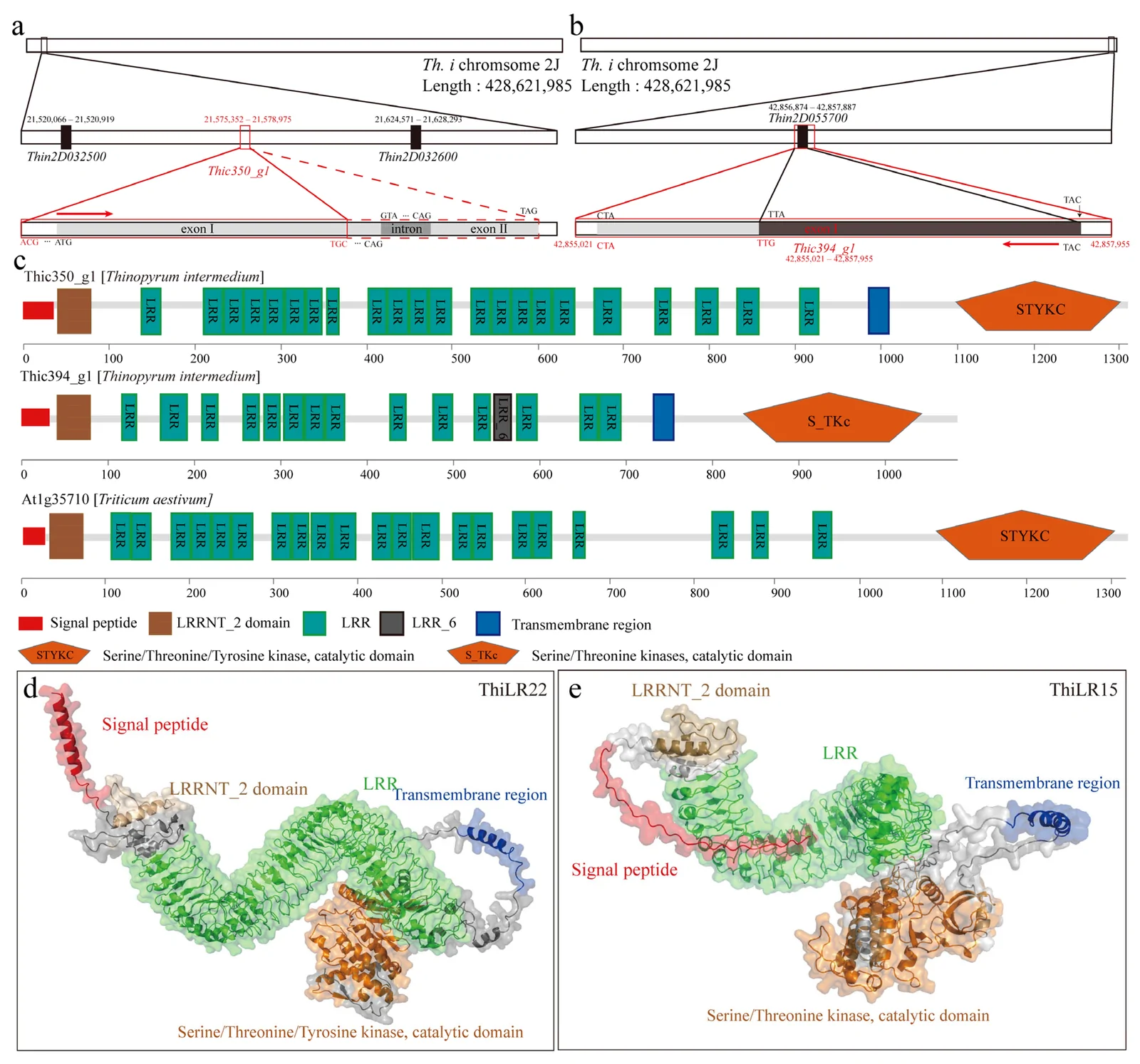
Chromosomal localization and structural characterization of two LRR-RLK candidate resistance genes derived from *Th. intermedium* chromosome 2J: (**a,b**) Chromosomal locations and exon–intron structures of *Thic350_g1* (*ThiLRR22*, **a**) and *Thic394_g1* (*ThiLRR15*, **b**). The red dashed line highlights the genomic region examined for exon structure at the transcript’s 3’ end. In **b**, the solid red and black lines represent the sequenced transcript and the annotated gene *Thin2D055700*, respectively. Red bases represent *Thic394_g1*, whereas black bases represent *Thin2D055700*. Arrows and minus signs indicate antisense-strand orientation. The annotated TAA stop codon corresponds to CAA in the candidate transcript, extending its open reading frame. (**c**) Schematic comparison of conserved functional domains in ThiLRR22, ThiLRR15 and the wheat protein XP_044318967.1, annotated as probable leucine-rich repeat receptor-like protein kinase At1g35710. Colors denote the domains shown in the legend. (**d**,**e**) Predicted three-dimensional structural models of ThiLRR22 and ThiLRR15 proteins, with domain coloring consistent with panel (**c**).

Two candidate genes belonging to subcluster G-C1 of Cluster 6, namely *Thic350_g1* and *Thic394_g1*, were characterized via sequence alignment and genome annotation. *Thic350_g1* is located on the plus strand of *Thinopyrum intermedium* chromosome 2J, within the intergenic interval between annotated loci *Thin2D032500* and *Thin2D032600*. It contains two exons and encodes a 1341-amino-acid (aa) LRR-RLK (leucine-rich repeat receptor-like kinase) protein predicted to carry 22 tandem leucine-rich repeat (LRR) motifs; we designated this gene *ThiLRR22* (Figure 7a). *Thic394_g1* fully spans the annotated gene *Thin2D055700* (genomic position: 42856874–42857887), plus an extra 1853 bp of upstream flanking sequence and 68 bp of downstream flanking sequence without overlapping adjacent annotated genes. Sequence comparison revealed that *Thic394_g1* possesses a longer coding sequence (CDS) than the official annotation of *Thin2D055700*. This single-exon gene produces a 1096 aa LRR-RLK protein with 15 predicted tandem LRR motifs, hereafter designated ThiLRR15 (Figure 7b). Both ThiLRR22 and ThiLRR15 differ structurally from the wheat LRR-RLK protein At1g35710 (Figure 7c), indicating they represent two novel LRR-RLK members originating from *Th. intermedium* (Figure 7d,e).

Collectively, these findings suggest that the introgressed *Th. intermedium* chromosome coordinate MAPK signaling and core plant–pathogen interaction pathways during wheat–*Bgt* interplay. This study provides promising candidate genes for developing *Th. intermedium*-specific molecular markers and future map-based cloning of key resistance genes.

Additionally, multiple *Th. intermedium*-derived transcripts responsive to *Bgt* infection were identified in SN6307’s leaf transcriptome data, including resistance (*R*) genes, receptor kinase and signal transduction-related genes, and several non-coding RNAs (ncRNAs). These results confirm that these alien genes from *Th. intermedium* play important roles in responding to *Bgt* infection and provide candidate genes for the development of specific molecular markers for the *Th. intermedium* 2J chromosome.

### 2.7. Development of Molecular Markers for the Identification of Chromosome 2J

**Figure 8:**
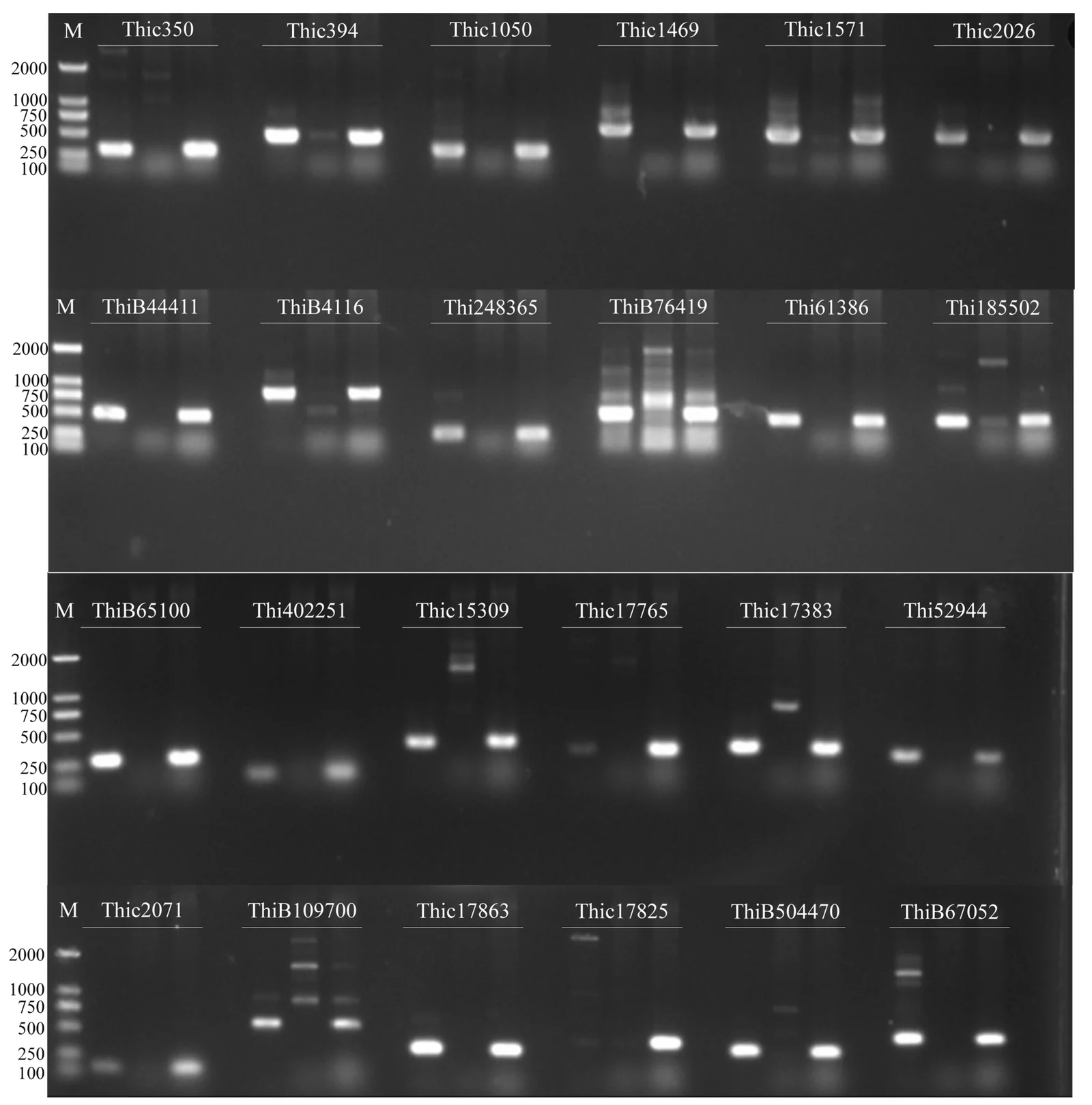
DNA marker analysis of SN6307, YN15 and *Th. intermedium.* M: DNA ladder (DM2000), with the 750 bp band labeled. For each group (marked with a white underline), the lanes from left to right represent the PCR amplification results using genomic DNA from SN6307, YN15 and *Th. intermedium*, respectively.

To enable rapid identification of wheat materials harboring *Th. intermedium* chromosome 2J and construct a physical map of functional molecular markers on this chromosome, we developed 24 primer pairs for molecular markers based on the PacBio third-generation full-length de novo transcriptome and Illumina second-generation de novo transcriptome data from SN6307. Specifically, these primers amplified target bands of consistent size only in materials containing the 2J chromosome, including the wheat-*Th. intermedium* 2J(2D) substitution line SN6307 and *Th. intermedium*, whereas no target bands were detected in the recurrent parent YN15 (Figure 8). Subsequently, homologous gene alignment and putative protein annotation were performed for the transcripts corresponding to these molecular markers, and the results showed that 3 markers were derived from receptor kinase-related transcripts, 4 markers were from transcripts annotated as mRNAs associated with disease resistance, transport and metabolism, 4 markers were from transcripts annotated as non-coding RNAs and mRNAs encoding other functional proteins, and 13 markers were from unannotated transcripts (Table S2). Collectively, these developed markers provide reliable tools for the rapid identification of chromosome 2J, and will lay a solid foundation for the subsequent isolation of key functional genes via radiation mutagenesis and hybridization, as well as the creation of wheat germplasm with desirable agronomic traits.

## 3. Discussion

### 3.1. The Th. intermedium Genome as a Source for Improving Wheat Disease Resistance and Agronomic Traits

Cultivated wheat possesses a narrow genetic base and uniform genetic backgrounds across cultivars. These characteristics, coupled with the continuous adaptive evolution of pathogenic fungi, have gradually eroded the effectiveness of conventional wheat disease resistance genes. Introgression of favorable genes from wheat wild relatives through distant hybridization has become a core strategy to broaden wheat genetic resources and improve comprehensive agronomic performance [28]. *Th. intermedium* is widely recognized as a tertiary gene pool for enhancing wheat genetic diversity. Its complex genome structure and exceptional stress resistance make it indispensable in chromosome engineering for wheat breeding. According to the latest genomic research, the genome formula of *Th. intermedium* is defined as JJVVStSt [9]. The J subgenome exhibits extremely high genetic similarity to *Aegilops* genomes, which may explain the strong interspecific compatibility between *Th. intermedium* and wheat, as well as its extensive application in wheat genetic improvement programs [9]. This allopolyploid structure endows *Th. intermedium* with robust environmental adaptability, and its disease resistance and stress tolerance genes can broaden wheat genetic resources following intergeneric introgression.

Accumulated cytogenetic and phenotypic evidence has confirmed that *Th. intermedium*-derived chromatin can enhance common wheat’s disease resistance, yield, and grain quality. A series of elite wheat-*Th*. *intermedium* alien lines have been developed, and their breeding value has been demonstrated. For disease resistance, the disomic alien addition line TA3681 (1St) and translocation line TA5566 (3A–7J) confer effective resistance to prevalent stem rust pathogens in China and Australia, respectively, providing valuable resistance resources for rust prevention [29]. The small-segment translocation line 4StL-4DL exhibits high resistance to stripe rust and powdery mildew, carrying novel resistance genes derived from *Th*. *intermedium* [30]. For yield improvement, the 6DS.6J^vs^ translocation line WT153397 displays increased effective tiller number and grain yield with stable inheritance, owing to the complete functional compensation of the wheat 6DL chromosome arm by the 6J^vs^ fragment; subsequent progeny populations have further optimized its grain size defect, verifying the favorable breeding potential of *Th. intermedium* J-subgenome chromosomes [31].

The T2DS·2V#4L translocation line carrying *Dasypyrum villosum* 2V#4L exhibits high adult-plant resistance to sharp eyespot, with stable genetic transmission of the translocation chromosome. Introgression of this translocation into common wheat increases productive spike number and spike length without adversely affecting other agronomic traits [32]. Existing studies have revealed that homoeologous group 2 chromosomes of wheat carry abundant genes governing multiple agronomic traits such as plant height and grain weight [33,34], and adverse agronomic performance occurs in certain translocation lines. For instance, the wheat–rye T2BS·2RL translocation leads to delayed heading and maturity in wheat [35]. Our study demonstrates that the *Th. intermedium* 2J chromosome exhibits excellent genetic compensation when substituting the wheat 2D chromosome. Introgression of the 2J chromosome significantly increases spike length in wheat, and the resulting line outperforms its recurrent parent YN15 and the widely cultivated cultivar JM22 in key quality parameters including grain protein content, sedimentation value, dough stability time and dough extensibility, with outstanding comprehensive grain quality performance. Collectively, these findings highlight the value of *Th. intermedium* introgression through substitution, addition, or translocation for improving disease resistance and agronomic traits in wheat. In the present study, SN6307 exhibited high resistance to *Bgt* isolate *E09* in detached-leaf assays at the seedling stage.

### 3.2. Characterization and Application Potential of the Wheat-Th. intermedium 2J(2D) Substitution Line SN6307

In line with these previous reports, our team previously identified the wheat-*Th. intermedium* disomic alien addition line SN6306, which exhibits near-immunity to the *Bgt* physiological race *E09*, and cloned the key alien powdery mildew resistance gene *TiAP1* from its alien chromosome [15]. Building on this foundation, the present study further characterized a genetically stable wheat-*Th. intermedium* 2J(2D) disomic alien substitution line SN6307. Disease resistance assays demonstrated that SN6307 exhibits near-immunity to the prevalent *Bgt* race *E09*, with distinctly improved resistance compared to its recurrent parent YN15. Transcriptome analysis identified additional *Bgt*-responsive candidate genes derived from *Th. intermedium* chromosome 2J. These genes may contribute to resistance alongside *TiAP1*, but their resistance functions and relative contributions require genetic and functional validation.

In addition to its superior disease resistance, SN6307 exhibited prominent improvements in key yield-related agronomic traits, including spike length, grain length, and hundred-kernel weight, without obvious negative effects on other agronomic traits. Moreover, SN6307 also showed good seedling salt tolerance, with higher biomass than ShanRong3 and Chinese Spring after 7 days of treatment with 250 mmol/L NaCl. Grain quality evaluation confirmed that SN6307 outperformed both YN15 and JM22 in multiple critical quality traits, such as dry-basis protein content, dough stability time, and dough extension length. Collectively, SN6307 integrates excellent powdery mildew resistance, superior grain quality, favorable yield traits, and stable genetic performance, demonstrating its great potential as promising elite germplasm for wheat breeding.

To further exploit the alien chromosomal resources and break potential unfavorable genetic linkages, we conducted ^60^Co-γ radiation mutagenesis on SN6307 and SN6306, and abundant phenotypic variations including multiple highly powdery mildew-resistant lines were obtained in the mutagenized progenies. Further studies will combine GISH, FISH, and transcriptome sequencing technologies to systematically mine novel valuable genes from alien translocation and introgressed segments derived from *Th. intermedium*.

### 3.3. Transcriptome Analysis Reveals a Novel Strategy of Wheat in Response to Bgt Infection Under 2J Chromosome Substitution

The advancement of transcriptome sequencing technology has greatly enhanced our understanding of alien chromatin introgression in wheat. Studies on various alien addition lines, translocation lines, and introgression lines have consistently demonstrated that the introduction of alien chromatin into the wheat genome can trigger extensive genome-wide transcriptional reprogramming. These studies have successfully characterized the enhanced disease resistance, grain quality, and yield traits conferred by alien chromatin fragments, and multiple valuable alien genes (including *FHB1* and *TiAP1*) have been cloned and functionally validated. In this study, we performed transcriptome sequencing on the 2J(2D) disomic substitution line at key stages of *Bgt* infection. The results showed that the 2J(2D) chromosome substitution exerted minimal disruptive effects on the global transcriptome under non-inoculated conditions, indicating that the functions lost owing to the absence of the 2D chromosome were effectively compensated.

Recent studies have demonstrated that chloroplast immunity plays a vital role in plant defense responses. With the continuous identification of immune pathways associated with chloroplasts, chloroplasts are now recognized as a central hub for immune signaling within plant cells. They generate a variety of defense-promoting signals and also serve as a major target for pathogens [36]. Notably, another key finding was that, compared with the non-inoculated group, differentially expressed genes (DEGs) associated with chloroplast and plastid pathways were significantly more enriched in the highly powdery mildew-resistant line SN6307 at 12 hpi. Although the roles of chloroplasts and plastids in pathogen recognition and secondary defense signal transduction have been well documented [37,38], the strong up-regulation of these pathways suggests that SN6307 possesses a more elaborate disease resistance network mediated by enhanced pathogen perception and signal transmission. This synergistic signaling module may underpin the elevated resistance observed in this substitution line. In contrast, the susceptible parent YN15 exhibited weakened activation of these pathways and prematurely initiated the RNA modification pathway, implying that YN15 attempted to compensate for impaired defense responses through post-transcriptional regulation.

Furthermore, analysis of the temporal dynamics of DEGs in both materials revealed that SN6307 deployed a sequential and ordered defense strategy: defense began with signal perception and protein transport at the early infection stage, followed by large-scale metabolic reprogramming, and ultimately culminated in excellent resistance via cellular homeostasis maintenance and cell wall reinforcement after successful suppression of *Bgt* infection. We also identified multiple candidate alien resistance genes, which provide valuable genetic resources for subsequent functional mechanism verification and molecular breeding applications.

### 3.4. Rapid Identification of Alien Gene Markers by Transcriptome Sequencing Combined with Marker-Assisted Selection (MAS)

Marker-assisted selection (MAS) offers distinct advantages in detecting alien chromatin in wheat, effectively improving the efficiency and accuracy of wheat breeding programs [39]. DNA microarrays and high-throughput sequencing technologies have been widely applied to develop molecular markers specific to alien chromosomes in wheat genetic improvement [15,40]. Notably, in this study, we directly identified a substantial number of mature long transcripts derived from *Th. intermedium* that respond to *Bgt* infection by performing full-length transcriptome sequencing on *Bgt*-inoculated leaves of SN6307. This trait-driven (targeting *Bgt* resistance) full-length transcriptome sequencing approach provides a universal analytical tool for the rapid identification of wheat-alien chromatin introgression lines and the mining of key candidate genes, thereby accelerating the expansion and utilization of wheat genetic resources.

Furthermore, based on multiple reference genomes of *Th. intermedium*, we successfully developed 24 specific molecular markers for the *Th. intermedium* 2J chromosome. Alignment of homologous gene sequences and functional annotation were further performed on the transcripts corresponding to these markers, leading to the annotation of multiple *Th. intermedium*-derived disease resistance-related genes. Collectively, the development of these 2J chromosome-specific markers not only enriches the marker library for detecting alien chromatin in wheat but also facilitates the targeted introgression and traceability of elite traits (e.g., powdery mildew resistance) from *Th. intermedium* into the wheat genome, providing robust technical support for molecular breeding of disease-resistant wheat varieties.

## 4. Materials and Methods

### 4.1. Plant Materials and Pathogen Infection

The hexaploid *Trititrigia* line ShanNong6307 (SN6307), a wheat–*Th. intermedium* 2J(2D) disomic substitution line with high resistance to powdery mildew, was identified among the advanced-generation selfed progeny of the wheat–*Th. intermedium* disomic alien addition line SN6306. SN6306 was previously developed through hybridization between common wheat cultivar YanNong15 (YN15, highly susceptible to *Bgt*) and *Th. intermedium* and has been described in earlier studies [15,41]. In the present study, the chromosome constitution of SN6307 was characterized using GISH, ND-FISH, Oligo-FISH painting and SNP array analysis. Seeds of all test materials were sown in a 1:1 (*v*/*v*) mixture of substrate and vermiculite, and cultured in a greenhouse under controlled conditions: 25 °C, 70% relative humidity, 16 h light/8 h dark long-day photoperiod, and a light intensity of 600 μmol/(m^2^·s). The highly virulent *Bgt* isolate *E09* (kindly provided by Professor Hongjie Li from the Institute of Crop Sciences, Chinese Academy of Agricultural Sciences) was propagated on YN15 via weekly subculture inoculation. All materials are preserved in the State Key Laboratory of Wheat Improvement, Shandong Agricultural University (Taishan District, Tai’an City, Shandong Province, China).

### 4.2. GISH, ND-FISH and Oligo-FISH Painting Analyses

Metaphase chromosome spreads of germinated seed root tips were prepared according to the protocol described by Han et al. [42]. Oligonucleotide probes (Oligo-pSc119.2, Oligo-pTa535, Oligo-B11, Oligo-K288, and Oligo-(GAA)_7_) were synthesized for ND-FISH [23,25,27,43,44], with their 5′ ends labeled with 6-carboxyfluorescein (6-FAM, green signal) or tetramethylrhodamine (Tamra, red signal). The sequences of the Oligo probes were taken from the report of Jiang et al. [45]. The labelled oligo probes were diluted in 1× Tris–EDTA (TE) buffer (pH 7.0), and 8 μL of probe mixture (containing 2× saline–sodium citrate (SSC), 1× TE buffer (pH 7.0), and each probe) was added to cell spreads, covered with coverslips, and incubated in a humid chamber at 42 °C for 40 min. Slides were then washed with 2× SSC at room temperature. Following ND-FISH, Oligo-FISH painting was performed as described by Li and Yang [17]. All probe sequences are listed in Table S1.

For GISH, metaphase chromosome spreads were prepared as described above. Genomic DNA of *Th. intermedium* was labeled with ChromaTide Alexa Fluor 488-5-dUTP (Thermo Fisher Scientific, Eugene, OR, USA) as the probe, and YN15 genomic DNA was used as the blocking DNA. The probe was mixed with GISH buffer, and slides were denatured for 4 min 30 s, followed by hybridization at 42 °C for over 12 h. Sequential FISH was performed on the same slides using Oligo-pTa535 and Oligo-pSc119.2 probes according to standard procedures. Finally, chromosomes were counterstained with 4′,6-diamidino-2-phenylindole (DAPI, Invitrogen Biotechnology, Shanghai, China) and imaged using an Olympus BX-63 microscope (Tokyo, Japan).

### 4.3. Evaluation of Agronomic Traits

All materials were planted in the Agronomy Experimental Field of Shandong Agricultural University (Taishan District, Tai’an City, Shandong Province) during the 2024–2025 growing season. Flag leaf characteristics were investigated at the grain-filling stage, and population phenotypes, plant height, spike length, and other agronomic traits were recorded at maturity. Common wheat cultivars YN15 and Jimai22 (JM22) were used as controls. Grain quality traits were determined using a near-infrared quality analyzer (DA 7250, PerkinElmer, Waltham, MA, USA), with at least three biological replicates per material. Data are presented as the mean ± standard deviation (SD). Differences in agronomic and grain quality traits were assessed using one-way analysis of variance (ANOVA), with *p* < 0.05 considered statistically significant. Replicate numbers are specified in the corresponding figure legends and Supplementary Table S4.

### 4.4. Powdery Mildew Resistance Testing

Macro- and micro-phenotypes of *Bgt*-inoculated leaves of SN6307 and YN15 were analyzed using a modified detached leaf assay and a modified Coomassie Brilliant Blue (CBB) staining method. For the modified detached leaf assay: one-week-old wheat leaves were placed in Petri dishes lined with sterile non-woven fabric soaked in a 75 ppm benzimidazole solution [46], inoculated with fresh *Bgt* conidia, and cultured under the conditions described in Section 4.1 for phenotypic observation. For the modified CBB staining method: *Bgt* spores were inoculated via plate inoculation; leaf epidermal tissues were peeled off, soaked in 1% Triton X-100 for 30 min, rinsed in 1× phosphate-buffered saline (PBS) for another 30 min, and stained with Coomassie Brilliant Blue R-250 (CBB R-250). Detailed procedures are provided in Supplementary Methods. Data are presented as the mean ± SD from at least three biological replicates. Differences in lesion area and haustorium formation rate between SN6307 and YN15 were assessed using Student’s t-test, with *p* < 0.05 considered statistically significant.

### 4.5. Salt Tolerance Evaluation

Salt tolerance was evaluated following the method described by Qi et al. [47]. The salt-tolerant cultivar ShanRong3 and the salt-sensitive cultivar Chinese Spring (CS) were used as controls. Test seeds were soaked in deionized water until germination, and 3-day-old germinated seeds were transferred to 96-well hydroponic boxes containing Hoagland nutrient solution supplemented with 250 mmol/L NaCl (solution replaced twice a week). Cultivation conditions were the same as those described in Section 4.1. After 7 days, seedling growth was recorded. The activities of peroxidase (POD), superoxide dismutase (SOD), and catalase (CAT) in leaves were determined following the methods described by Nawaz et al. [48] to evaluate physiological differences among the tested materials. Antioxidant enzyme activity data are presented as the mean ± SD from at least three biological replicates. Differences among wheat lines were assessed using one-way ANOVA, with *p* < 0.05 considered statistically significant.

### 4.6. 55K Wheat SNP Array Analysis

The 55K wheat SNP array contains ~55,000 SNP markers evenly distributed across 21 wheat chromosomes (an average of ~2600 markers per chromosome, an average genetic distance of ~0.1 cM and a physical distance < 300 Kb between adjacent markers). Young embryos of SN6307 and YN15 were collected with a surgical blade, placed in 2 mL centrifuge tubes, snap-frozen in liquid nitrogen, and sent to Zhongyu Jinbiao (Beijing) Biotechnology Co., Ltd. (Beijing, China) for DNA library construction and probe hybridization. Raw data were subjected to strict quality control, and filtered reads were aligned to the reference genome using BWA (Burrows–Wheeler Aligner) software (v 0.7.18) to obtain high-quality SNP locus information. Data visualization and result presentation were performed using R software (v 4.4) packages.

### 4.7. Transcriptome Sequencing and Data Analysis

At the two-leaf-one-heart stage, SN6307 and YN15 were inoculated with *Bgt* spores via the modified plate inoculation method. Approximately 200 mg of leaf epidermal tissue from the inoculated area was collected at 0 (uninoculated), 12, 24, and 48 h post-inoculation (hpi), with three biological replicates per time point. Transcriptome sequencing was performed on the Illumina platform by Personalbio Technology Co., Ltd., (Shanghai, China). Raw sequencing data were filtered using FASTX-Toolkit (0.0.14) to obtain clean reads, which were then aligned to the Chinese Spring V2.1 reference genome.

Mixed epidermal tissue samples of SN6307 (0, 12, 24, and 48 hpi) were subjected to de novo sequencing on the PacBio platform, with correction using Illumina-based full-length transcriptome sequencing data to obtain information on *Bgt*-induced transcripts in SN6307. From the Illumina assembly results, the longest transcript of each gene was selected as a unigene for gene naming and functional annotation. Genes with fragments per kilobase of transcript per million mapped reads (FPKM) ≥ 1 were subjected to differential expression analysis using DESeq2 software (v 1.44.0), with screening criteria of |log_2_FoldChange| ≥ 1 and adjusted *p*-value ≤ 0.05. Gene Ontology (GO) functional annotation and Kyoto Encyclopedia of Genes and Genomes (KEGG) pathway enrichment analysis were performed to explore the biological functions of differentially expressed genes (DEGs).

### 4.8. Development of Specific Molecular Markers for Th. intermedium

Third-generation PacBio full-length transcriptome data of SN6307 were aligned to the Chinese Spring V2.1 reference genome using NCBI BLAST locally (v2.15.0). Transcripts labeled “Not Found” or meeting the threshold (Cover < 90% or Ident < 90%) were retained, and further BLAST analysis was performed using WheatOmics (https://wheatomics.sdau.edu.cn//) to select transcript sequences potentially derived from *Th. intermedium* chromosomes (reference genomes: Chinese Spring CS-IAAS T2T [49] and *Th. intermedium* PI440031 chromosomes [9]).

Specific primers for candidate transcripts were designed using NCBI Primer-BLAST (reference species, Taxonomy ID: 4565) and synthesized by Sangon Biotech (Shanghai, China). DNA from all materials was extracted from seedling leaves using the cetyltrimethylammonium bromide (CTAB) method [23]. The specificity and validity of the markers were verified using SN6307, YN15, and *Th. intermedium*. Twenty-four primer pairs produced *Thinopyrum*-specific bands and their sequences are listed in Table S2. Each 20 μL PCR reaction contained 10 μL of 2 × 3G Taq Master PCR Mix (Vazyme Biotech Co., Ltd., Nanjing, China), 40–100 ng genomic DNA, and each primer at a concentration of 0.2 mmol/L. The amplification procedure was as follows: initial denaturation at 94 °C for 5 min, followed by 34 cycles of denaturation at 94 °C for 30 s, annealing at the temperature specified for the corresponding primers for 45 s, and extension at 72 °C for 1 min. A final extension was performed at 72 °C for 10 min. The PCR products were detected and analyzed by 1% agarose gel electrophoresis.

### 4.9. Protein Structure Prediction Using AlphaFold

AlphaFold3 (v3.0.1) [50] was deployed locally at the High-Performance Computing Center at Shandong Agricultural University. Monomeric protein structures were predicted using AlphaFold3. A fixed seed value of 100 was applied to ensure reproducibility. Homologous sequence data used to generate multiple sequence alignments (MSAs) were collected from six databases: UniRef90, MGnify, BFD, PDB, Uniclust30, and Swiss-Prot [51]. Then, MSAs generated by JackHMMer (3.4) and HHblits (3.3.0) provided evolutionary inputs for AlphaFold modeling. PyMOL(v 2.6) was used to visualize the three-dimensional protein structures. Domain analysis was performed by submitting target protein sequences to the SMART online server, and domain characteristics were analyzed based on the predicted protein structures.

## 5. Conclusions

This study characterizes SN6307, a novel wheat–*Th. intermedium* 2J(2D) disomic substitution line, which exhibits near-immunity to powdery mildew at the seedling stage while retaining desirable agronomic traits. Integrated cytogenetic and multi-omics analyses reveal that *Th*. *intermedium*-derived LRR-RLK genes and pathogen-response pathways coordinately orchestrate a multilayered immune network in SN6307. Additionally, 24 new 2J-specific molecular markers were developed for efficient chromatin tracking. SN6307 thus provides valuable disease-resistant germplasm and a foundation for cloning functional genes from wild relatives to combat powdery mildew in wheat breeding.

## Data Availability

The original contributions presented in this study are included in the article. Further inquiries can be directed to the corresponding authors.

## Supplementary Materials

The following supporting information can be downloaded at: https://www.mdpi.com/article/10.3390/plants15193061/s1, Figure S1. Distribution of SNP_Na from the 55K wheat SNP array within 1 Mb sliding windows across all chromosomes in YN15 and SN6307. Figure S2. Transcriptome correlation analysis of leaf samples from SN6307 and YN15 at different time points post-*Bgt* inoculation. Figure S3. Analysis of differentially expressed genes induced by corresponding *Bgt* infection in wheat materials. Figure S4. Analysis of differentially expressed patterns in SN6307 upon powdery mildew infection. Table S1. The sequences of Oligo probes used in this study. Table S2. The developed marker sequences for 2J chromosomes in *Th. intermedium* species. Table S3. The proportion of missing loci among the total SNP loci on each chromosome. Table S4. Determination of hundred-kernel weight. Table S5. Key pathway genes and annotation information of Cluster 6. Method S1. Modified CBB R-250 staining method.
