# Supplementary material for: Molecular Cytogenetic and Transcriptome Characterization of a Novel Wheat-Thinopyrum intermedium 2J(2D) Disomic Substitution Line with Resistance to Powdery Mildew
Source: Plants (Basel). 2026 Oct 7;15(19):3061. doi: 10.3390/plants15193061 (PMC13667210; doi:10.3390/plants15193061)
Supplement: Supplementary file 1 [file plants-15-03061-s001.zip › plants-4534630-supplementary.pdf]

Supplementary Files

Figure S1. Distribution of SNP_Na from the 55K wheat SNP array within 1-Mb sliding windows across all chromosomes in YN15 and SN6307.

Figure S2. Transcriptome correlation analysis of leaf samples from SN6307 and YN15 at different time points post *Bgt* inoculation.

Figure S3. Analysis of differentially expressed genes induced by corresponding *Bgt* infection in wheat materials.

Figure S4. Analysis of differentially expressed patterns in SN6307 upon powdery mildew infection.

Table S1. The sequences of Oligo probes used in this study.

Table S2. The developed marker sequences for 2J chromosomes in *Th. intermedium* species.

Table S3. The proportion of missing loci among the total SNP loci on each chromosome.

Table S4. Determination of hundred-kernel weight.

Table S5. Key pathway genes and annotation information of Cluster 6.

Method S1. Modified CBB R-250 staining method.

a

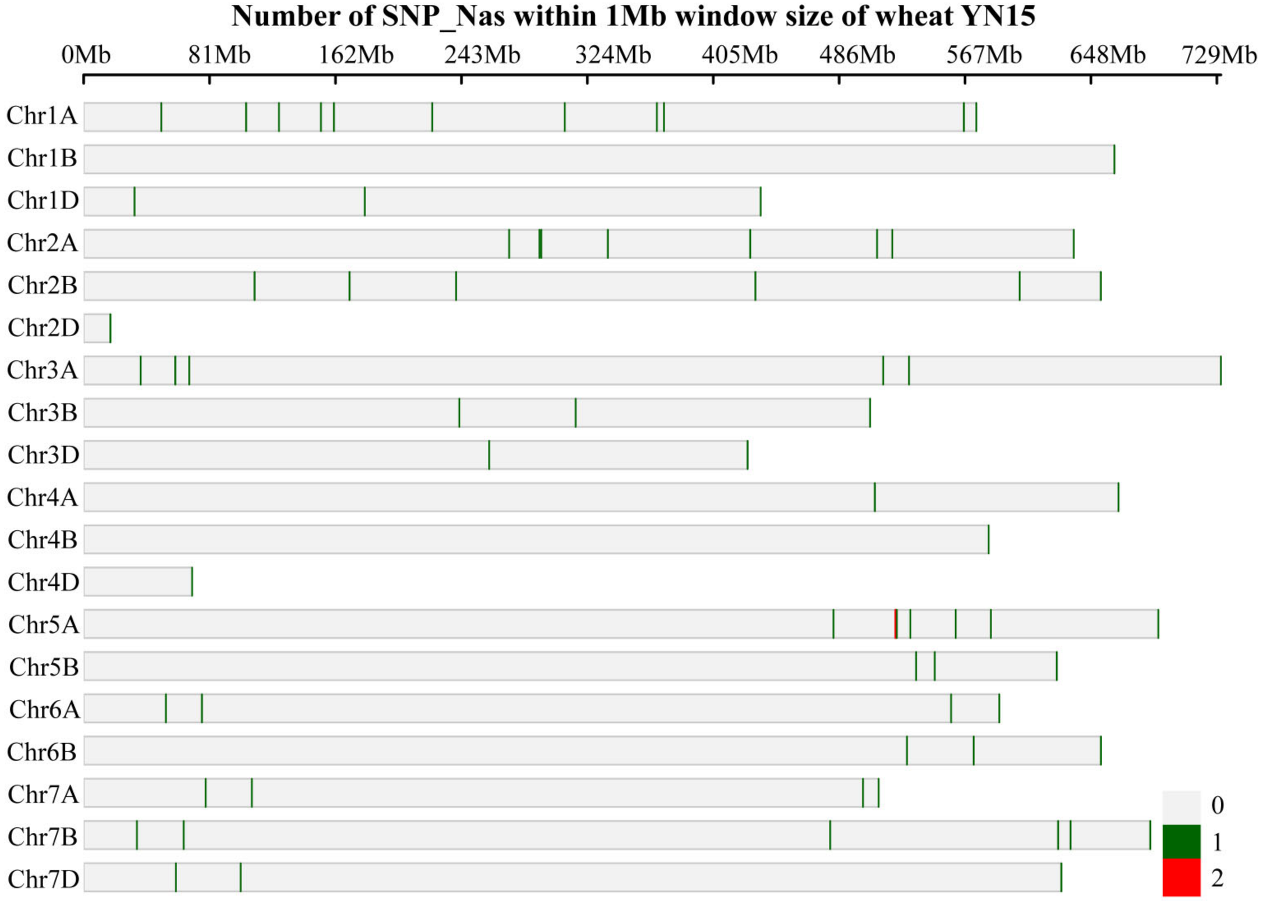

b

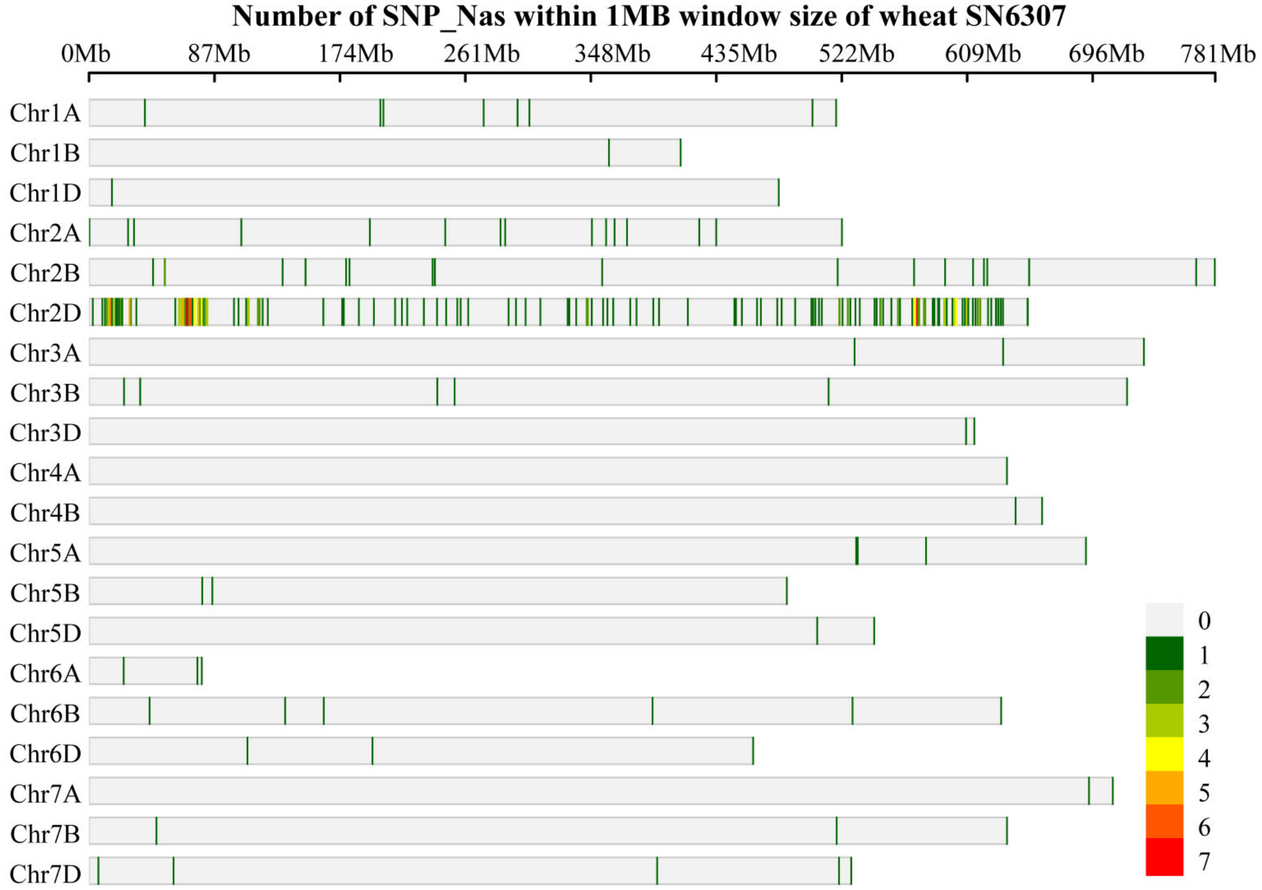

Figure S1. Distribution of SNP_Na from the 55K wheat SNP array within 1-Mb sliding windows across all

chromosomes in YN15 and SN6307. Distribution of SNP_Na detected by the 55K wheat SNP array within 1-Mb sliding windows across all chromosomes in YN15 (a) and SN6307 (b). Colors indicate the number of missing SNP calls within each 1-Mb window, as defined by the respective legends. Chromosomes with no missing SNP calls are omitted.

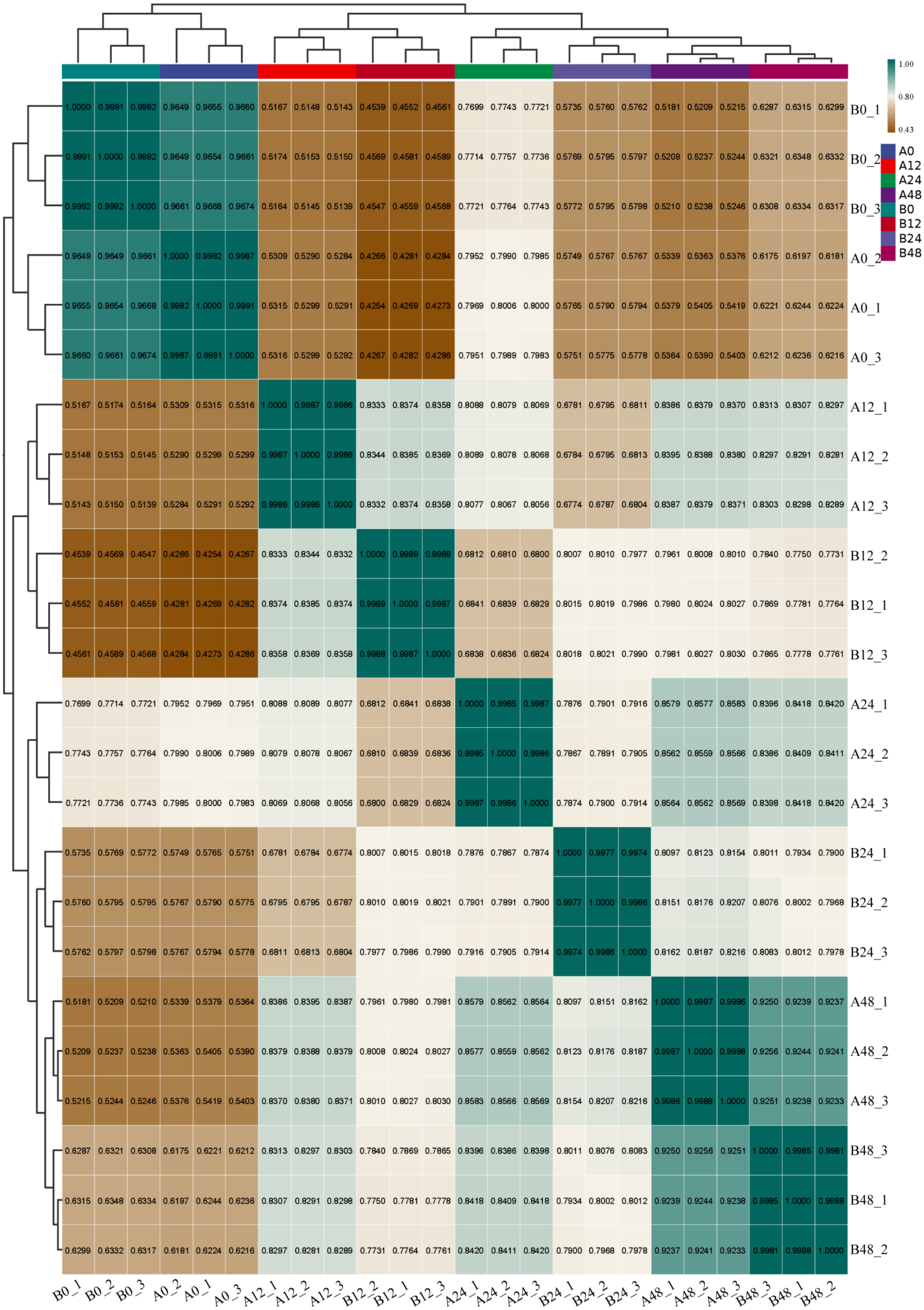
1.00
0.80
0.43
A0
A12
A24
A48
B0
B12
B24
B48
B0_1 1.0000 0.9991 0.9992 0.9649 0.9655 0.9660 0.5167 0.5148 0.5143 0.4539 0.4552 0.4561 0.7699 0.7743 0.7721 0.5735 0.5760 0.5762 0.5181 0.5209 0.5215 0.6287 0.6315 0.6299
B0_2 0.9991 1.0000 0.9992 0.9649 0.9654 0.9661 0.5174 0.5153 0.5150 0.4569 0.4581 0.4589 0.7714 0.7757 0.7736 0.5769 0.5795 0.5797 0.5209 0.5237 0.5244 0.6321 0.6348 0.6332
B0_3 0.9992 0.9992 1.0000 0.9661 0.9668 0.9674 0.5164 0.5145 0.5139 0.4547 0.4559 0.4568 0.7721 0.7764 0.7743 0.5772 0.5795 0.5798 0.5210 0.5238 0.5246 0.6308 0.6334 0.6317
A0_2 0.9649 0.9649 0.9661 1.0000 0.9982 0.9987 0.5309 0.5290 0.5284 0.4266 0.4281 0.4284 0.7952 0.7990 0.7985 0.5749 0.5767 0.5767 0.5339 0.5363 0.5376 0.6175 0.6197 0.6181
A0_1 0.9655 0.9654 0.9668 0.9982 1.0000 0.9991 0.5315 0.5299 0.5291 0.4254 0.4269 0.4273 0.7969 0.8006 0.8000 0.5765 0.5790 0.5794 0.5379 0.5405 0.5419 0.6221 0.6244 0.6224
A0_3 0.9660 0.9661 0.9674 0.9987 0.9991 1.0000 0.5316 0.5299 0.5292 0.4267 0.4282 0.4286 0.7951 0.7989 0.7983 0.5751 0.5775 0.5778 0.5364 0.5390 0.5403 0.6212 0.6236 0.6216
A12_1 0.5167 0.5174 0.5164 0.5309 0.5315 0.5316 1.0000 0.9987 0.9986 0.8333 0.8374 0.8358 0.8088 0.8079 0.8069 0.6781 0.6795 0.6811 0.8386 0.8379 0.8370 0.8313 0.8307 0.8297
A12_2 0.5148 0.5153 0.5145 0.5290 0.5299 0.5299 0.9987 1.0000 0.9986 0.8344 0.8385 0.8369 0.8089 0.8078 0.8068 0.6784 0.6795 0.6813 0.8395 0.8388 0.8380 0.8297 0.8291 0.8281
A12_3 0.5143 0.5150 0.5139 0.5284 0.5291 0.5292 0.9986 0.9986 1.0000 0.8332 0.8374 0.8358 0.8077 0.8067 0.8056 0.6774 0.6787 0.6804 0.8387 0.8379 0.8371 0.8303 0.8298 0.8289
B12_2 0.4539 0.4569 0.4547 0.4266 0.4254 0.4267 0.8333 0.8344 0.8332 1.0000 0.9989 0.9988 0.6812 0.6810 0.6800 0.8007 0.8010 0.7977 0.7961 0.8008 0.8010 0.7840 0.7750 0.7731
B12_1 0.4552 0.4581 0.4559 0.4281 0.4269 0.4282 0.8374 0.8385 0.8374 0.9989 1.0000 0.9987 0.6841 0.6839 0.6829 0.8015 0.8019 0.7986 0.7980 0.8024 0.8027 0.7869 0.7781 0.7764
B12_3 0.4561 0.4589 0.4568 0.4284 0.4273 0.4286 0.8358 0.8369 0.8358 0.9988 0.9987 1.0000 0.6838 0.6836 0.6824 0.8018 0.8021 0.7990 0.7981 0.8027 0.8030 0.7865 0.7778 0.7761
A24_1 0.7699 0.7714 0.7721 0.7952 0.7969 0.7951 0.8088 0.8089 0.8077 0.6812 0.6841 0.6838 1.0000 0.9985 0.9987 0.7876 0.7901 0.7916 0.8579 0.8577 0.8583 0.8396 0.8418 0.8420
A24_2 0.7743 0.7757 0.7764 0.7990 0.8006 0.7989 0.8079 0.8078 0.8067 0.6810 0.6839 0.6836 0.9985 1.0000 0.9986 0.7867 0.7891 0.7905 0.8562 0.8559 0.8566 0.8386 0.8409 0.8411
A24_3 0.7721 0.7736 0.7743 0.7985 0.8000 0.7983 0.8069 0.8068 0.8056 0.6800 0.6829 0.6824 0.9987 0.9986 1.0000 0.7874 0.7900 0.7914 0.8564 0.8562 0.8569 0.8398 0.8418 0.8420
B24_1 0.5735 0.5769 0.5772 0.5749 0.5765 0.5751 0.6781 0.6784 0.6774 0.8007 0.8015 0.8018 0.7876 0.7867 0.7874 1.0000 0.9977 0.9974 0.8097 0.8123 0.8154 0.8011 0.7934 0.7900
B24_2 0.5760 0.5795 0.5795 0.5767 0.5790 0.5775 0.6795 0.6795 0.6787 0.8010 0.8019 0.8021 0.7901 0.7891 0.7900 0.9977 1.0000 0.9986 0.8151 0.8176 0.8207 0.8076 0.8002 0.7968
B24_3 0.5762 0.5797 0.5798 0.5767 0.5794 0.5778 0.6811 0.6813 0.6804 0.7977 0.7986 0.7990 0.7916 0.7905 0.7914 0.9974 0.9986 1.0000 0.8162 0.8187 0.8216 0.8083 0.8012 0.7978
A48_1 0.5181 0.5209 0.5210 0.5339 0.5379 0.5364 0.8386 0.8395 0.8387 0.7961 0.7980 0.7981 0.8579 0.8562 0.8564 0.8097 0.8151 0.8162 1.0000 0.9987 0.9986 0.9250 0.9239 0.9237
A48_2 0.5209 0.5237 0.5238 0.5363 0.5405 0.5390 0.8379 0.8388 0.8379 0.8008 0.8024 0.8027 0.8577 0.8559 0.8562 0.8123 0.8176 0.8187 0.9987 1.0000 0.9988 0.9256 0.9244 0.9241
A48_3 0.5215 0.5244 0.5246 0.5376 0.5419 0.5403 0.8370 0.8380 0.8371 0.8010 0.8027 0.8030 0.8583 0.8566 0.8569 0.8154 0.8207 0.8216 0.9986 0.9988 1.0000 0.9251 0.9238 0.9233
B48_3 0.6287 0.6321 0.6308 0.6175 0.6221 0.6212 0.8313 0.8297 0.8303 0.7840 0.7869 0.7865 0.8396 0.8386 0.8398 0.8011 0.8076 0.8083 0.9250 0.9256 0.9251 1.0000 0.9985 0.9981
B48_1 0.6315 0.6348 0.6334 0.6197 0.6244 0.6236 0.8307 0.8291 0.8298 0.7750 0.7781 0.7778 0.8418 0.8409 0.8418 0.7934 0.8002 0.8012 0.9239 0.9244 0.9238 0.9985 1.0000 0.9988
B48_2 0.6299 0.6332 0.6317 0.6181 0.6224 0.6216 0.8297 0.8281 0.8289 0.7731 0.7764 0.7761 0.8420 0.8411 0.8420 0.7900 0.7968 0.7978 0.9237 0.9241 0.9233 0.9981 0.9988 1.0000
B0_1 B0_2 B0_3 A0_2 A0_1 A0_3 A12_1 A12_2 A12_3 B12_2 B12_1 B12_3 A24_1 A24_2 A24_3 B24_1 B24_2 B24_3 A48_1 A48_2 A48_3 B48_3 B48_1 B48_2

Figure S2. Transcriptome correlation analysis of leaf samples from SN6307 and YN15 at different time points post *Bgt* inoculation. Cell colors and values indicate Pearson correlation coefficients, and the top color bar identifies genotype–time combinations. A and B denote SN6307 and YN15, respectively; 0, 12, 24 and 48 denote sampling times in hours post-inoculation (hpi), with 0 representing the non-inoculated control. Suffixes _1, _2 and _3 identify the three biological replicates.

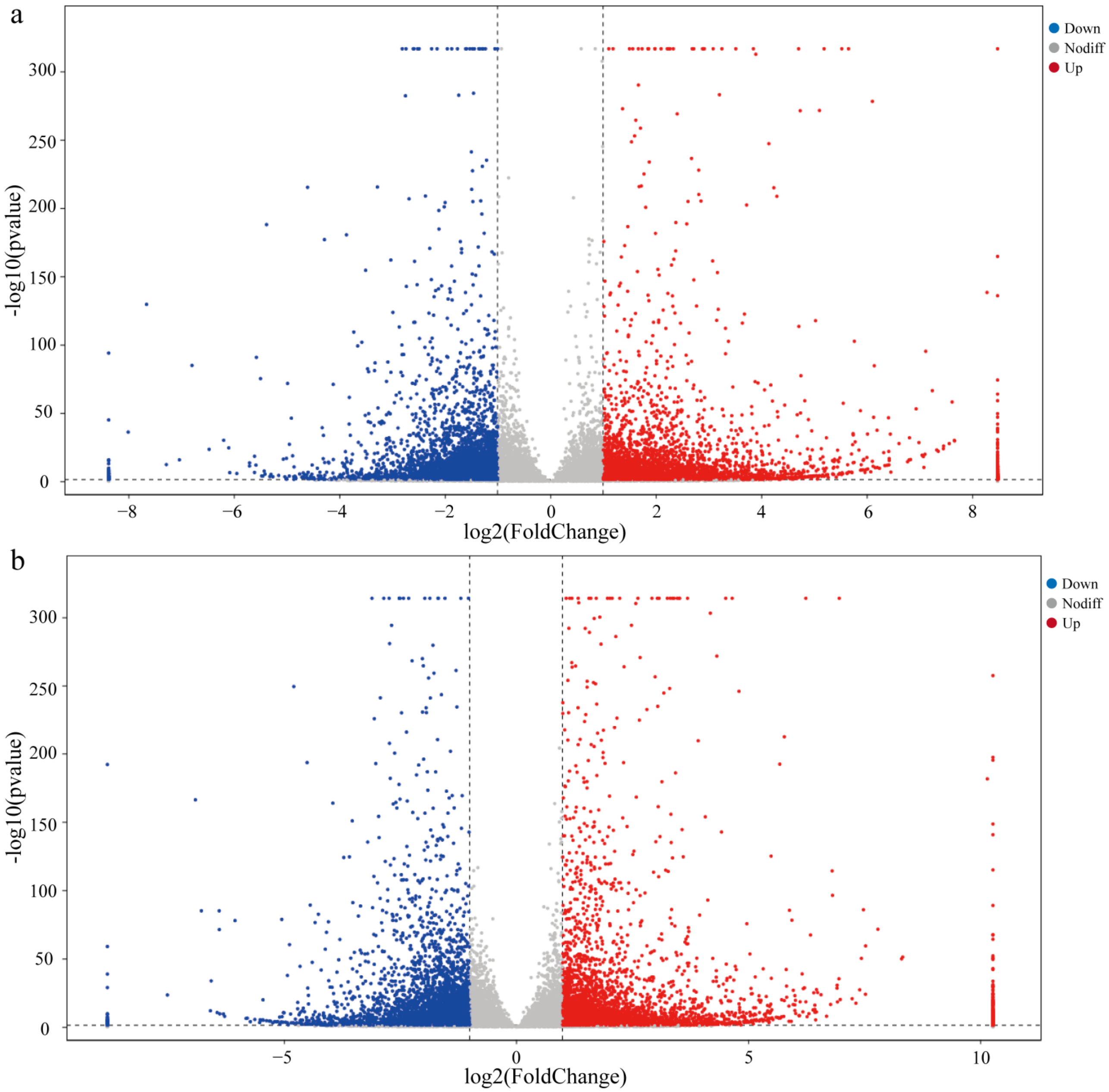

Figure S3. Analysis of differentially expressed genes induced by corresponding *Bgt* infection in wheat materials. Differential gene expression (DGE) analysis was performed using the DESeq2 software for SN6307 (a) and YN15 (b) samples at 12 hpi with *Bgt* conidia, using the non-inoculated group as the control. DEGs were screened with the criteria of |log2FoldChange| ≥ 1 and adjusted *P*-value ≤ 0.05. Volcano plots were used to display the DEGs, with red and blue dots representing up-regulated and down-regulated DEGs. Gray dots indicate genes that do not meet the differential-expression criteria. Dashed lines mark the thresholds used.

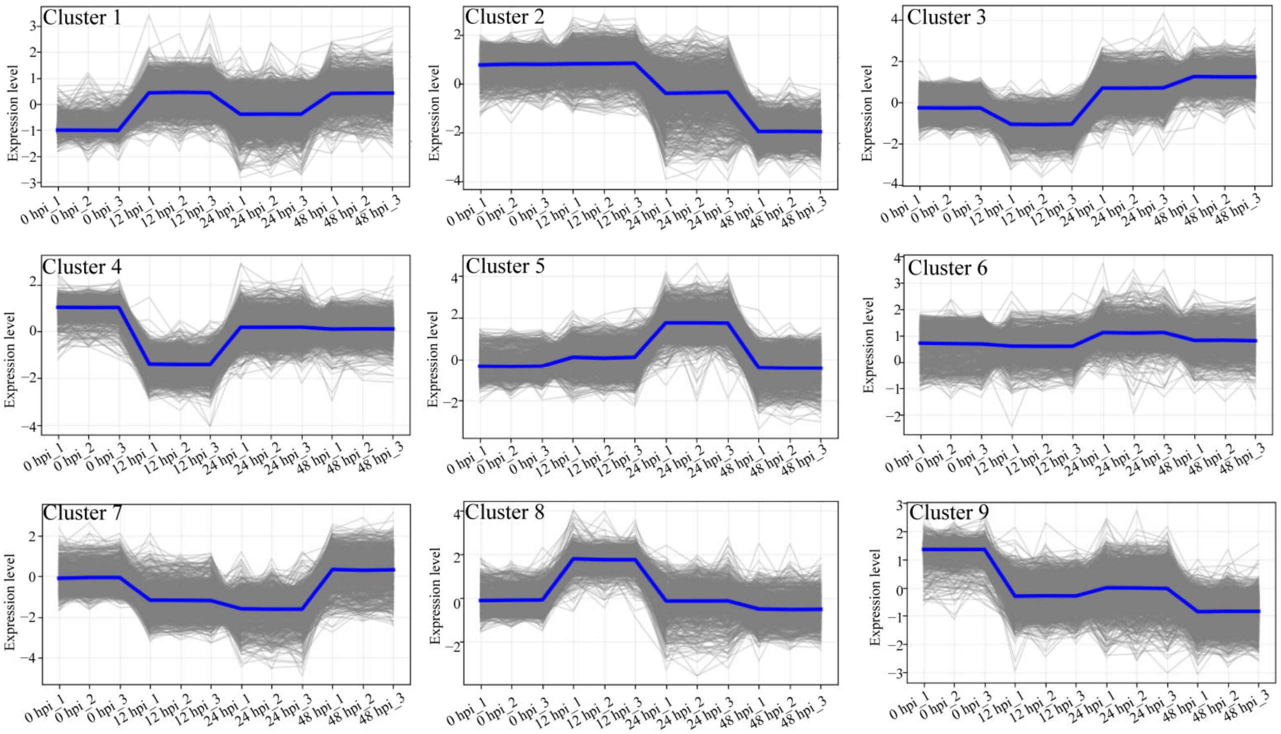

Figure S4. Analysis of differentially expressed patterns in SN6307 upon powdery mildew infection. All full-length transcripts were grouped into nine distinct clusters (Cluster 1 to Cluster 9) based on their dynamic expression profiles along the infection course. Thin gray lines represent the expression trajectory of individual transcripts within each cluster, while solid blue lines indicate the mean expression trend of all transcripts in the corresponding cluster. The x-axis shows samples collected at 0, 12, 24 and 48 hours post-inoculation (hpi), with 0 denoting the non-inoculated control and suffixes _1, _2 and _3 indicating biological replicates. The y-axis shows expression values standardized as Z-scores for each transcript.

Table S1. The sequences of Oligo probes used in this study

Oligo probes	Sequences (5’ - 3’)	Reference
Oligo-pTa535	AAAAACTTGACGCACGTCACGTACAAATTGGACAAACTCTTTCG GAGTATCAGGGTTTC	Tang et al. 2014 [25]
Oligo-pSc119.2	CCGTTTTGTGGACTATTACTCACCGCTTTGGGGTCCCATAGCTAT	Tang et al. 2014 [25]
Oligo-B11	TCCGCTCACCTTGATGACAACATCAGGTGGAATTCCGTTCGAGGG	Xi et al. 2019 [23]
Oligo-K288	CTTCATAGTCCGGGAGTCCGGCCAAAGGTCATAGTCCGGCCATCC	Wang et al. 2019 [43]
Oligo-$(GAA)_7$	GAAGAAGAAGAAGAAGAAGAA	Feng et al. 2020 [27]

Table S2. The developed marker sequences for 2J chromosomes in *Th. intermedium* species

ID	Forward	Reverse	Annotation of associated transcripts	Melting temperature (℃)
Thic350	AGAACAACCTCACCGGTTCC	ACCCAGGAGTCTATTGCTGC	leucine-rich repeat receptor-like protein kinase mRNA	60.0 / 58.3
Thic394	GCGAAATCTCAACCGAGGGA	GGCAACGGAAAAGATAGCAAGT	LRR receptor-like serine/threonine-protein kinase mRNA	60.0 / 60.1
Thic1050	CTAGAATCGAGCGCAACGCA	CCGTCGACATAATGCTCATCC	-	59.1 / 60.3
Thic1469	TAGCCTTCTGCAGAGTTGCC	TAAACGCTGCTCCCCTCTTG	noncoding	58.6 / 59.2
Thic1571	AAGAAGCTCTGCAAGATGGGC	GGGAATCATCCTCCACCCAC	NBS-LRR disease resistance protein	61.8 / 60.9
Thic2026	ATCATGCCCGATAGTGGTCG	TTACGCTCCTCGCTTACACAT	metal-nicotianamine transporter YSL9-like mRNA	58.6 / 60.0
ThiB44411	AATCACCGCAATGCCTCA	AATCCGTATCTCGTATCTACTCTGT	receptor homology region, transmembrane domain- and RING domain-containing-like mRNA	56.4 / 58.2
ThiB4116	TCTCTCGCGGAAGTCTCGT	GCTTCATTATCAGCGGTGG	-	60.1 / 55.5
Thi248365	CGGAGAAGAATGGACAAGG	CGTTTCACCCCATG	-	55.8 / 49.8
ThiB76419	AGTCGTCGCCGTCCTCCA	CTACACCGGCATCGACGT	beta-fructofuranosidase, insoluble isoenzyme 7-like mRNA	63.5 / 59.2
Thi61386	GAGATAATTGCGGCACATCG	GAGGACCCATAGGCTAGTTGA	-	55.8 / 60.3
Thi185502	CAACCAAGCCCAGGAAAT	GCAAAGGAGACGGCAAGT	-	55.3 / 57.6
ThiB65100	GCAGACCACCTAATTCTCAT	GAGACGATGGTAAGAGGATG	-	53.6 / 54.3
Thi402251	CCTAACCAAGTGAGACATCGG	TGCTGTACCCTGGAGAACG	-	60.2 / 58.9
Thic15309	TCGCTCACCAGCCAGAATAC	TTTTAGACCCGCCTCTGCTC	noncoding	58.7 / 59.1
Thic17765	AGCCAGTGCTCTCGTTTTGT	TCCTCAGATCTGGAAGGCCA	-	58.3 / 60.1
Thic17383	TAATGGCAGTTTGGGGTGCT	CCCTCACTTCGTATTCGCCA	-	58.5 / 59.3
Thi52944	GCGAGATCCACCACCACAGA	CTCCAGGGCGAAAACAAAGG	-	61.6 / 59.4
Thic2071	CCTAGCAGCAGCAGCAAGTAT	ATGACTCCCGAGAGGGAAGA	FACT complex subunit SPT16-like mRNA	60.1 / 59.8
ThiB109700	TCATGGCGCTGGAGCAT	TGAGGGATCACGAAGGAT	-	58.3 / 55.1
Thic17863	TGATCTGTGCTGTGGCTGAG	AACTGCCTCCCTTTAGGCAC	*Leymus chinensis* salt-induced protein 3-like mRNA	58.6 / 59.1
Thic17825	TAGTCGGTGCATCCCCTTCT	CTTCGCACCATGACGAACAC	-	59.9 / 58.8
ThiB504470	GAGCCACTCTTTACCTTCG	CCTTGATGAGCACCATCC	G-type lectin S-receptor-like serine/threonine-protein kinase At2g19130-like mRNA	55.2 / 55.3

ThiB67052	GGCTGTAGCACACTAATC	CTTGCAACCATGTCACTG	-	51.5 / 53.3

Primer sequences are shown in the 5′–3′ direction. Paired melting-temperature values correspond to the forward and reverse primers, respectively.

Table S3. The proportion of missing loci among the total SNP loci on each chromosome

Genome Group	Chromosome	Missing Ratio (%) of YN15	Missing Ratio (%) of SN6307
Group A	1A	0.647820966	0.471142521
	2A	0.470035253	0.881316099
	3A	0.405131668	0.202565834
	4A	0.109469075	0.054734537
	5A	0.470865215	0.235432607
	6A	0.248911014	0.186683261
	7A	0.241837969	0.120918984
Group B	1B	0.069444444	0.069444444
	2B	0.390370852	0.130123617
	3B	0.177830468	1.126259632
	4B	0.05608525	0.336511497
	5B	0.171330668	0.114220445
	6B	0.186915888	0.373831776
	7B	0.357995227	0.178997613
Group D	1D	0.220588235	0.147058824
	2D	0.138312586	30.84370678
	3D	0.426439232	0.426439232
	4D	0.138888889	0.000000000
	5D	0.000000000	0.179051030
	6D	0.000000000	0.257289880
	7D	0.220264317	0.367107195
Whole Genome	-	Mean: 0.245;<br>Median: 0.220;<br>Standard Deviation: 0.173	Mean: 1.748;<br>Median: 0.203<br>Standard Deviation:6.672

Missing Ratio (%) of each chromosome = (Number of Na_SNPs / Total SNP number on the corresponding chromosome) × 100%

Table S4. Determination of hundred-kernel weight

Replicate	SN6307 hundred-kernel weight (g)	YN15 hundred-kernel weight (g)	ShanRong 3 hundred-kernel weight (g)	JM22 hundred-kernel weight (g)
1	3.3	3.1	4.4	5.1
2	3.4	3.2	4.3	5.2
3	3.2	3.2	4.2	5.1
4	3.3	3.1	4.3	5.0
5	3.4	3.3	4.3	5.1

Each replicate consisted of 100 randomly sampled kernels. Five replicate samples were weighed for each wheat line. JM22, Jimai22.

Table S5. Key pathway genes and annotation information of Cluster 6

Gene ID	Source	Annotation of associated transcripts
c9953_g1	*Th. intermedium*	Mitogen-activated protein kinase kinase kinase 1b-like
c7953_g1	*Wheat* & *Th. intermedium*	OPAQUE10
c10335_g1	*Th. intermedium*	Calcium-Dependent Protein Kinase, CDPK/CPK
c2043_g1	*Th. intermedium*	HSP83
c8902_g1	*Th. intermedium*	Glycerol kinase
c5771_g1	*Th. intermedium*	ETHYLENE-INSENSITIVE 3-like 2
c17036_g1	*Th. intermedium*	Basic endochitinase C-like
c10451_g1	*Wheat &Th. intermedium*	Catalase-1-like
c394_g1	*Th. intermedium*	LRR receptor-like serine/threonine-protein kinase
c17042_g1	*Wheat* & *Th. intermedium*	26 kDa endochitinase 1-like
c1240_g1	*Th. intermedium*	Ethylene receptor 2
c13421_g1	*Wheat* & *Th. intermedium*	Serine/threonine-protein kinase SAPK5
c350_g1	*Th. intermedium*	Leucine-rich repeat receptor-like protein kinase
c10909_g1	*Wheat* & *Th. intermedium*	Mitogen-activated protein kinase kinase kinase 1b-like
c12873_g1	*Wheat* & *Th. intermedium*	Indolin-2-one monooxygenase
c4888_g1	*Th. intermedium*	LncRNA
c5805_g1	*Th. intermedium*	LncRNA
c5726_g1	*Th. intermedium*	LncRNA
c1228_g1	*Th. intermedium*	LncRNA
c9339_g1	*Th. intermedium*	LncRNA

Source: The online BLAST service of WheatOmics was adopted, with the reference genomes including PacBio Full Length cDNA (*Triticum aestivum*), Chinese Spring CS-IAAS T2T gene, Chinese Spring gene (RefSeq v2.1) and *Thinopyrum intermedium* PI440031. Functional annotation was conducted using NCBI blastp with default parameters against the Clustered NR (nr_cluster_seq) database.

Method S1. Modified CBB R-250 staining method

1. Preparation of wheat leaf epidermal tissues

Wheat seedlings were grown to the two-leaf-one-heart stage. An L-shaped acrylic plate was placed at the pot edge, with two 2-mm-wide double-sided tapes attached parallel to the bend. Leaves were fixed abaxial-side up on the plate and placed in an inoculation chamber. *Blumeria graminis* f. sp. *tritici E09* spores were evenly blown onto leaves from the top corners; the chamber was sealed for 30 min, followed by incubation at 25°C and 70% relative humidity.

At sampling, leaves were excised and fixed at the base. The leaf tip was incised to retain only lower epidermis, which was carefully peeled from mesophyll tissue, trimmed, and immersed in 1.5 mL of 1 × PBS buffer (2 mL tube).

2. Cell Component Elution

The PBS buffer was removed; samples were incubated in 1% Triton X-100 on a shaker for 30 min, then washed three times with 1 × PBS (10 min each) to elute interfering substances while preserving cell morphology.

3. Coomassie Brilliant Blue R-250 Staining

Samples were stained with 1.5 mL 0.025% Coomassie Brilliant Blue R-250 at room temperature for 2 h, rinsed three times with deionized water, mounted, and observed microscopically.
